# Presence of Arsenic in Potential Sources of Drinking Water Supply Located in a Mineralized and Mined Area of the Sierra Madre Oriental in Mexico

**DOI:** 10.3390/toxics9110307

**Published:** 2021-11-15

**Authors:** Victor Manuel Escot-Espinoza, Yann Rene Ramos-Arroyo, Isabel Lázaro, Isidro Montes-Avila, Leticia Carrizalez-Yañez, Roberto Briones-Gallardo

**Affiliations:** 1 Facultad de Ingeniería, Universidad Autónoma de San Luis Potosí, Av. Dr. Manuel Nava 304, San Luis Potosí 78210, Mexico; victor.escot@alumnos.uaslp.edu.mx (V.M.E.-E.); ilazaro@uaslp.mx (I.L.); isidro.montes@uaslp.mx (I.M.-A.); 2 Departamento de Ingeniería Geomática e Hidráulica, Universidad de Guanajuato, Av. Juárez 77, Zona Centro, Guanajuato 36000, Mexico; yr.ramos@ugto.mx; 3 Instituto de Metalurgia, Universidad Autónoma de San Luis Potosí, Sierra Leona 550, San Luis Potosí 78210, Mexico; 4 CIAAS/CIACYT, Facultad de Medicina, Universidad Autónoma de San Luis Potosí, Sierra Leona 550, San Luis Potosí 78210, Mexico; letcay@uaslp.mx

**Keywords:** mine wastes, secondary mineral phases, arsenic, dissolution mechanism, spring water quality

## Abstract

Mine wastes from the La Aurora mine in the state of Guanajuato were generated by the flotation process and placed in four tailing dumps on the local stream while the plant operated. Given that these wastes contain toxic elements, it is important to establish their impact on the quality of several surrounding natural sources of water that are considered potential drinking water supplies. This study identified four water source types, in which the contents of arsenic (As), mercury (Hg), and thallium (Tl) were exceeded, according to international guideline values for drinking water quality. The first type of aqueous sample corresponded to leachates produced by rainwater infiltration in tailings and water–mineral waste interactions. The second type corresponded to surface water along the Xichú and La Laja Streams, and the third and fourth types involved two groundwater well samples and spring samples, respectively. The Chiquito Stream was used as a reference area that had not been impacted by the mine wastes. The isotopic signatures associated with δ^34^S_sulfate_ and δ^18^O_sulfate_ compositions from the El Ojo de Agua spring are similar to those of the Santa María River and are different from those of the mine waste leachates. This study shows evidence of the presence of As in the El Ojo de Agua spring, which results from dissolution of secondary mineral phases that were produced by alteration of the mine wastes, which then migrated along the Xichú Stream system until reaching the spring. These As-bearing fine particles are prone to dissolution when in contact with this water source. Principal component analysis revealed that the observed As, Tl, and Hg can be attributed to weathering of the mine wastes. However, the results suggest that a natural contribution of these elements could be associated with rainwater–igneous rock interactions.

## 1. Introduction

For many years, mining and metallurgical activities have generated hundreds of millions of tons of waste. The types of generated waste include the tailings from several operations, such as flotation, amalgamation, cyanidation, or refining processes, as well as slags and dust particles from smelting furnaces [1]. Worldwide, tailings dumps are mainly composed of particles of different sizes that have been placed at sites adjacent to the mine processing area and represent many abandoned mine waste dumps (AMWDs) [2,3,4,5]. These AMWDs generally lack pollution control measures and are typically piled near abandoned mines. Waste dumps resulting from skarn-type mineral processing are characterized by pyrite (Py), marcasite (Mrc), galena (Gn), sphalerite (Sp), chalcopyrite (Ccp), covellite (Cv), arsenopyrite (Apy), argentite (Arg), aluminosilicates (Als), calcite (Cal), and other carbonate-rich rocks [6]. In the absence of adequate control and containment measures, these wastes undergo alteration processes (physicochemical or biological) when exposed to environmental conditions (precipitation, flood, evaporation, desiccation, solar radiation, drought, and wind) [4,7,8]. Some of these AMWDs that are rich in Pyrite produce acid mine drainage (AMD) via sulfide oxidation processes, and the produced leachates are typically highly acidic and have high concentrations of potentially toxic elements (PTEs), which primarily include arsenic (As), lead (Pb), mercury (Hg), and thallium (Tl) [1,7,9,10,11]. Hence, AMD neutralization generates new secondary mineral phases (SMPs) that contain large quantities of PTEs that are either adsorbed, precipitated, or coprecipitated [10]. Some secondary phases reported as products of AMD neutralization are melanterite (Mel), copiapite (Cpt), coquimbite (Cqm), ferricopiapite (Fcpp), schwertmannite (Swm), gypsum (Gy), anglesite (Ang), K-jarosite (K-Jrs), beudantite (Bdt), scorodite (Scr), ferrihydrite (Fhy), goethite (Gth), hematite (He), and lepidocrocite (Lpd) [4]. Some of the reactions that produce SMPs under such conditions have been described by several authors [1,7,12,13,14,15].

The main hypothesis of this study, which focuses on how PTEs are produced and impact water bodies, supposes that—depending on local climatological conditions and the topographical positions of the dumps—the new SMPs have the potential to react with the environment and remain in the waste dumps, thereby forming a stable cemented crust. However, solid fragments of these SMPs could disseminate and be incorporated into stream sediments. Once in the stream bed, SMPs have the potential to migrate downstream by hydric erosion, accumulate in low-energy areas such as floodplains, and continue their travels until they reach different bodies of water, thereby affecting the quality of the stream surface and groundwater. Consequently, the accumulation of As in water sources originates from geogenic or anthropogenic processes [16,17].

The finer PTE-bearing particles can move via suspension in surface water and infiltrate through the porous spaces of stream sediments [18,19]. Skarn deposits have ubiquitous occurrences of arsenopyrite [6]. In the environment, As can be found in two oxidation states, As(V) or As(III) (as oxyanions, arsenates, or arsenites). As(V) species are predominant under oxidizing conditions, while As(III) species predominate under reducing conditions, and their equilibrium speciation depends on the pH and the redox potential (E_H_) of the system [9,20,21]. Due to the slow oxidation–reduction rate of these species, inorganic chemical species of both As(III) and As(V) can coexist simultaneously. The As(V) leached from AMWDs can be attenuated via adsorption, precipitation, or coprecipitation involving iron oxides, iron oxide-hydroxides (IOH), and iron oxide-hydroxy-sulfates (IOH-SO_4_^2−^), such as ferrihydrite, goethite, and Jrs, among other SMPs [1,4,7].

Conversely, the presence of Hg is not exclusively associated with skarn deposits, as it is possible to find contents as high as 5000 and 9000 µg/kg in igneous and alkaline rocks, respectively [22,23]. High concentrations of Hg in surface waters (up to 100 ng/L) have been reported when natural waters are in contact with rocks or sediments with a high content of this element [24]. Recently, pollution of water with Tl, associated with AMD, has been reported. Thallium pollution in the public water supply system in a location in Italy was identified and associated with the draining of the spring throughout a mineralized pyritous zone with a high content of this element [25,26].

The region studied in this work is in a rugged, mountainous zone in the Sierra Gorda of Guanajuato, which has faced several challenges related to developing infrastructure that favors water accessibility. Based on a volume of 31.5 Mm^3^, that drains from springs annually from these sources [27], the drinking water supply for the Sierra Gorda marginalized communities can potentially be sustained. Therefore, it is important to determine the water quality and to describe the geochemical processes that control it. Hence, this work focuses on identifying the presence of As, Hg, and Tl and evaluating their impact on natural springs, well groundwater, and surface water. In addition, it is important to discern whether the presence of these elements is natural or associated with the leachates coming from the AMWDs at the now inactive mine known as La Aurora in the Xichú mining district (XMD).

## 2. Study Site Description

### 2.1. Geological and Hydrological Context

**Figure 1:**
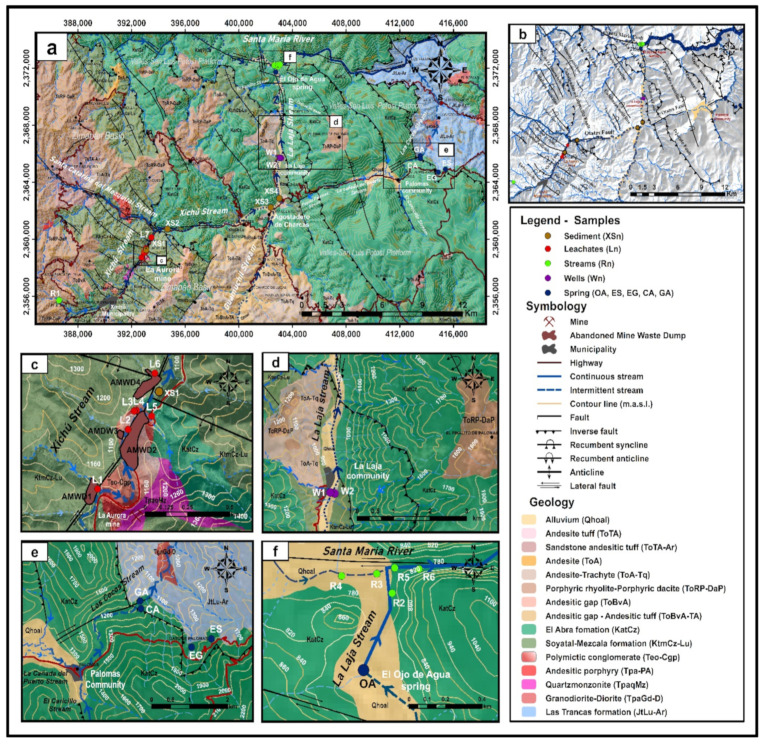
(**a**) Map of the study area and location of the collection of water and sediment samples. (**b**) Geological structural map representing the main fault, orography, and hydrography context. (**c**) Close-up view of the wastes area. (**d**) Close-up view of the La Laja community area. (**e**) Close-up view of the Palomas community area. (**f**) Close-up view of the confluence between the El Ojo de Agua spring and the Santa María River. The labels identify the sampling points by the type of stream body or the stream sediments, or both (based on data from the geological maps of Arredondo-Mendoza et al. [32] and González-Ramos and Rodríguez-Moreno [34]).

The XMD is in the Sierra Madre Oriental physiographic region in the subprovince of Karst-Huasteco in the Zimapán Basin (Figure 1a). According to the topographic map of the study area, the altitudes range from 2640 to 800 m above sea level (m.a.s.l.), with very abrupt slopes [27,28]. According to the Köeppen–Geiger climate classification, the study area is classified as Bsh, which corresponds to hot semi-arid (sttepe) climate [29].

From registers of a meteorological station situated 7 km southwest of the Xichu tailings in the same hydrological basin, it was found that in this zone predominates orographic rainfall that discharges as storms of high intensity. A climatograph (Figure S1), constructed with the average of monthly data from the period 1951–2015 [30], shows that the period of rains is between spring and summer and the average annual precipitation is 575.5 mm, characterized by a midsummer drought or heatwave, during July–September. Almost every year, a storm of 20 mm in one hour occurs, and the maximum of daily precipitation ever registered is 158 mm [31]. All the materials in this basin are exposed to the effect of erosivity of rains. Particularly, tailings deposits can be eroded. The erosion effect depends on the layer crust formed on the surface of the mine wastes.

The AMWDs were placed on the Xichú Stream hillside over the Soyatal–Mezcala Formation (KtmCz-Lu). Quartz monzonitic intrusions (TpaQMz) are distributed in this formation close to the La Aurora and El Cristo mines, heading NW–SE and affecting the terrigenous sediments of the Soyatal–Mezcala Formation. This gives rise to two types of mineralized alterations: the Xichú alteration and the Tijeras-Peña Bernal-Lucero-San Diego alteration. The mineralized bodies of the Xichú alteration are in a contact halo shape, forming exoskarns with structures such as veins, mantles, and irregular chimneys [32]. According to the Mexican geological survey [32], the skarn mineralized bodies exploited in La Aurora mine have their origin from hydrothermal solutions at moderate–high temperatures, which are characteristic of epithermal and mesothermal deposits [33]. The Tijeras-Peña Bernal-Lucero-San Diego alteration surrounds the La Aurora mine on its NW and SE flanks and is housed in the El Abra (Kat-Cz) and Soyatal–Mezcala Formations (Figure 1a). This alteration is mainly composed of iron oxides produced by the oxidation of pyriterites, which are found filling fractures [32].

The XMD is in the hydrological region of the Santa María Bajo River subbasin (RH26Cj) that is part of the Panuco River, which discharges at the Gulf of Mexico.

Due to the geological characteristics of this zone, three types of aquifers can be found: fractured mediums associated with Tertiary rhyolites, granular mediums in recent alluvial valleys, and a karstic medium in the limestones of the El Abra Formation [27]. This formation is a geological structure characterized by high permeability, due to karstic conditions that, combined with faults and fractures, promote the discharge of various springs in the study area.

The Chiquito Stream is considered a reference area that has not been impacted by the AMWDs from the XMD. The background values of As, Hg, and Tl concentrations in the water within the study area were established based on a sample of the Chiquito Stream (location R1, Figure 1), which is located close to the El Alamo community at 1634 m.a.s.l., which is 8.5 km upstream of the area with mine waste dumps and has a confluence with the Xichú Stream after the Misión de Santa Rosa (Figure 1a). This stream outcrop is in a geological zone that favors fractures in the igneous porphyritic rhyolite–porphyritic dacite (ToRP-DaP) (Figure 1a). This type of volcanic rock can be associated with hydrothermal alteration and can contribute to the presence of toxic elements such as As, Hg, and Tl [8,22,24,25]. A second reference area that has also not been impacted by AMWDs is connected to four springs that discharge close to the Palomas community at ~1300 m.a.s.l. and 23 km to the northeast of the AMWDs from the XMD (Figure 1d), without any apparent hydraulic connection. This is a zone where springs and rainwater collection are a source of water and is geologically characterized by the contact between Cretaceous El Abra limestones and Jurassic shales of the Las Trancas Formation (Figure 1d). However, the use of spring water could be compromised due to the presence of As, Hg, and Tl. The limit for As and Hg concentrations were established by the World Health Organization (WHO) at 10 and 6 µg/L, respectively [35], and at 25 and 1 µg/L, respectively, by the Mexican normative [36]. With respect to Tl, the Environmental Protection Agency (EPA) has established a limit of 13 µg/L for the protection of human health [37].

The hydrogeology in this zone has not been thoroughly studied until now. To begin to develop a hydrological model, it is necessary to know that this region is located between two Cretaceous paleobasins: the Zimapan Basin to the west and the Valles-San Luis Potosí platform to the east [38]. The lithologies of both basins consist primarily of platform limestones with interbedded sandstones, and both have Jurassic shales of the Las Trancas Formation as the hydrogeological basement.

At the location where the Xichú Stream receives the Adjuntas Creek, As concentrations in the stream sediments reportedly range from ~13 to 40 mg/kg [32,34]. Approximately 10 km beyond this point is the beginning of the alluvial valley, with an accumulation of fine particles overlying gravel and sands. Due to the high permeability of this coarse material, the groundwater in local flow systems can circulate. The El Ojo de Agua spring is located on the La Laja Stream bed 7 km away from the La Laja community (Figure 1a) and is parallel to the La Laja lateral fault. The flow of this spring has been estimated to be between 850 and 1000 L/s [27,39]. This spring is influenced by both the Las Trancas Formation, which acts as the hydrogeological basement (locally mineralized), and the El Abra Formation. In this area, rainwater infiltrates the karstified material of the El Abra Formation [40], which could give rise to two groundwater wells used by the La Laja community or even the El Ojo de Agua spring (Figure 1e).

### 2.2. Origin and Characteristics of Abandoned Mine Waste Dumps

In the XMD, six abandoned mines have been recorded: La Aurora, Don Pedro, Lucero, El Cristo, Casa Blanca, and La Tijera [33]. The AMWDs evaluated in this study are located 4 km NE of the community of Xichú, Guanajuato, Mexico. There are a few reports that describe the mining residues associated with the activities of these mines, but these reports do not examine either the presence of PTEs in the neighboring soils or the Xichú Stream sediments that are derived from mine waste oxidation [27,41,42]. In fact, only one study has evaluated the environmental impact of the PTEs present in leachate from mine waste and the presence of these PTEs in some springs in the region [11]. Even though the La Aurora mine had multiple operational periods, this study considered the AMWDs generated during the period between 1935 and 1957.

Activities during this period involved the processing of skarn-type polymetallic sulfides that contained galena (3.5% Pb), sphalerite (5% Zn), chalcopyrite (2% Cu), Arg (which contains 250–750 g As/t), and pyrite [33]. The AMWDs are constituted by sulfide mineral phases with a pyrite–galena–sphalerite–chalcopyrite–covellite–hematite paragenetic sequence [43]. The ore mineral deposits that were processed were emplaced in limestone and sedimentary rocks [43,44,45]. After 1957, the mine and mine tailings were abandoned without any containment or prevention measures to mitigate their oxidation. Carrillo-Chávez et al. [41] evaluated the concentrations of the four AMWDs present on the banks of the Xichú Stream, finding As concentrations from 1753 to 62,302 mg/kg, as well as Pb concentrations (from 670 to 17,426 mg/kg), but they did not report Hg and Tl values. Also, they indicated that As and Pb pollution from wastes is attenuated in situ due to sorption, precipitation, or coprecipitation reactions in the dumps. Ramos-Arroyo et al. [27] estimated that the volumes of two sulfide-rich mine waste deposits located on the banks of the Xichú Stream were 41,973.55 m^3^ and 189,934.93 m^3^. These authors suggested that approximately 1 million tons of waste was generated from the flotation process during the recovery of lead (Pb), zinc (Zn), copper (Cu), and silver (Ag), and they suggested that the As release rate from AMWDs of the La Aurora tailings to the Xichú Stream bed is 2 kg/year.

Understanding As, Hg, and Tl dispersion and release mechanisms toward potential sources of water constitutes a crucial part of the contributions of this research. Likewise, it is important to associate springs with different groundwater flow systems to determine potential vulnerabilities to changes in weather and the presence of mineralization and mine wastes [46].

Geochemical maps from the Mexican Geological Survey (SGM) allow the identification of the average and maximum concentrations of As in this area: the average basal As concentration is ~8 ± 6 mg/kg (*n* = 215) in stream sediments, and the maximum As concentration ranges from 106 to 135 mg/kg [32,34]. The SGM does not report Hg or Tl concentrations in stream sediments in these geochemical maps.

In this work, evidence of the impact of As, Hg, Tl, and Pb on water bodies is provided. It has been verified through field inspection that detachment of SMPs occurs in large fragments due to hydric erosion and that these fragments are then incorporated into the channel of the Xichú Stream. These events show the physical entry of the contaminated fragments and the dispersion of PTEs bearing solid particles into the channel of the Xichú Stream. Additionally, rainstorm events allow these materials to move away from the source and migrate to low-energy areas, eventually impacting the studied water bodies, affecting their quality, and putting the water security of the area at risk [8].

## 3. Materials and Methods

### 3.1. Water Sampling and Chemical Analysis

The map in Figure 1 shows the physiographic provinces of the study area and the spatial location of the different collected samples. Different sources of water samples were collected and grouped as (1) leachates, (2) well water, (3) river water, and (4) spring water (Table 1).

Water samples were taken from the same site in three clean polypropylene containers. The determination of several parameters was performed as follows: (1) physicochemical parameters (temperature, pH, E_H_, electrical conductivity (EC), and dissolved oxygen (DO)); (2) total major and trace element concentrations (Na, Mg, K, Ca, Fe, Mn, Al, As, Pb, Cu, Zn, Sr, Mn, Hg, and Tl); and (3) anion concentrations (HCO_3_^−^, CO_3_^2−^, Cl^−^, SO_4_^2−^, and NO_3_^−^). All samples were filtered using 0.45 µm nylon filters prior to their respective analyses. Physicochemical parameters were determined in the field with multiparameter equipment (HANNA HI9828 with a Ag/AgCl reference electrode). All total major and trace element analyses were performed on the filtered samples to 0.2 µm using nylon filters and were acidified with 37% HNO_3_ using inductively coupled plasma atomic emission spectrometry (ICP-AES iCAP 7000, Thermo Scientific, Cambridge, United Kingdom. The As concentration was determined using a graphite furnace, coupled with an atomic absorption spectrometer (EAA-HG, Analyst 200 PinAAcle 900T, Perkin Elmer, Whaltham, United States). The reference material, NIST-1643f, was used as an internal control with an As concentration of 57.4 ± 0.4 μg/L and a recovery percentage of 99.6%. Anion determination was performed on filtered samples that were not acidified using anion exchange chromatography equipment (Dionex ICS-5000+, Thermo Scientific, San Jose, United State). In liquid samples, the alkalinity was determined in 100 mL of aqueous sample and 0.02 N H_2_SO_4_ for acid titration using phenolphthalein and methyl orange indicators, following Method 2320-A Alkalinity from the Standard methods [47]. The isotopic composition of δ^34^S_sulfate_ ‰ and δ^18^O_sulfate_ ‰ in SO_4_^2−^ ions was determined at the Environmental Isotopy Laboratory of the Department of Geosciences at the University of Arizona. The stable isotope of δ^34^S_sulfate_ ‰ in SO_4_^2−^ ions was reported with respect to the Devil’s Canyon Troilite (CDT) standard, while δ^18^O_sulfate_ ‰ in SO_4_^2−^ ions was reported with respect to Vienna standard mean ocean water (VSMOW).

### 3.2. Waste and Sediment Mineralogical Characterization

Six solid waste samples were recovered from AMWDs, three of them from the terrace of the deposit and the other three from the sidewalls where leachate drainage was observed. Additionally, four recent stream sediment samples were collected within an area of 1 m^2^ and at a depth of 5 cm. All sediment samples were collected in zones of low energy in the dry streambed at the time of sampling (XS1–XS4). All solid samples (wastes and sediments) were free of vegetal matter, sieved in the field using 2 mm mesh and transported to the laboratory in clean polyethylene bags. Subsequently, the samples were dried at room temperature for 72 h. The As concentrations in stream sediments were determined by X-ray fluorescence (XRF) using a portable analyzer (INNO-X DELTA, Olympus DPO-2000-CC). A mineralogical characterization of the stream sediments was accomplished by X-ray diffraction (XRD) using X-ray diffractometer (D8 Advance, Brucker, Berlin, Germany). XRD analysis was performed on samples with a particle size < 44 μm using the powder diffraction method with a source of Cu Kα radiation between 10 and 90°, a 2θ variation of 0.02° and an analysis total time of 21 min. X-ray diffraction pattern interpretation of stream sediments was performed by comparing the spectra identified with Joint Committee on Powder Diffraction Standards (JCPDS) cards from the International Center for Diffraction Data (ICDD). XRD analysis identified the mineral phases that make up the matrix or the PTE carrier phases, or both. To identify the presence of primary and As-bearing SMPs, mineralogical characterization was complemented with scanning electron microscopy (SEM) using a Philips XL30 microscope equipped with an energy dispersive X-ray spectrometer (EDAX DX460), to perform quantitative chemical analyses.

### 3.3. Hydrogeochemical Analysis

**Figure 2:**
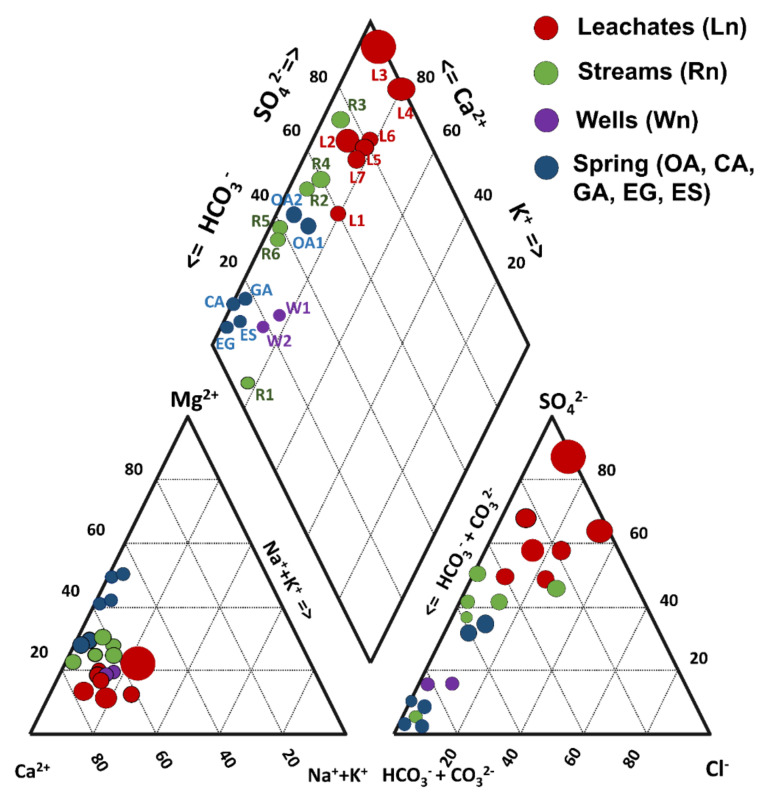
Piper diagrams for the different types of water present in the study area. The size of the marker increases as the EC concentration in the sample increases.

The major anion and cation concentrations in the water samples were used to generate Stiff and Piper diagrams using AquaChem 3.7 software (Waterloo hydrogeologic, Waterloo, ON, Canada). Since EC is considered to be the main chemical marker related to pyrite oxidation and AMD production [48], a Piper diagram was constructed by considering the EC value to normalize the size of the icon of the represented samples (Figure 2). For each aqueous solution sample, the charge balance was calculated using the WATEQ4F database in PHREEQC v. 2.15 [49], and the charge imbalance (CI) was calculated according to Nordstrom et al. [50]. Likewise, the saturation indices (SI) of mineral phases identified by XRD in the mine wastes were calculated using PHREEQC with the Thermoddem database [51]. Relationships between the physicochemical variables analyzed in the water samples were evaluated using Pearson’s correlation tests and principal component analysis (PCA). Pearson’s correlation was used to evaluate the relationship among physicochemical parameters, cations, and anions that were quantified in the leachates, streams, wells, and spring water samples in the study area. The correlation was also used to suggest the reactions driving the presence of major and trace elements in solution for all water samples. Table S1 shows that red and blue numbers have significant positive and negative correlations, respectively (*p* < 0.05). In the PCA, the eigenvalues and variances were determined to establish principal components (PCs). With PCA diagrams, it is possible to observe the interaction between the variables and samples; a grouping of samples suggests a common source [52].

Prior to PCA, all data measured in aqueous solution were transformed using a power function, b_ij_ = x_ij_^p^, with *p* = 0.5. Additionally, two inclusion criteria were used for the chemical and physicochemical variables: first, their values should be above the detection limit of the chemical method used, and second, the value of the variable should be at least 20% of samples. In addition, Fe, Cu, Pb, and Zn concentrations were incorporated and considered geochemical markers of mine waste weathering.

For the interpretation of the PCA results, both the eigenvectors of the first and second components were projected, as well as the graphic representation of the sample types, to identify the trends of the water flows. In this study, a PCA value was calculated for each of the aqueous samples [53]. In this case, up to five main components were considered, representing 82% of the covariance of samples. Hence, the dimension of the system was reduced from 25 to 5 transformed variables that correspond to the Z_isk_ scores. The calculation of the Z_isk_ scores was carried out with Equation (1) for each sample, as follows:

Z_isk_ = ∑_j_^m^ Ev_ij_ × var_j_^1/2^, (1)

where Ev_ij_ and var_j_ represent the values of the eigenvectors and the variables transformed to the square root power function, respectively.

With the value of the Z_isk_ scores, it was possible to calculate the PCA value for each of the samples (PCA_sk_) according to Equation (2), where Eg_i_ represents the eigenvalue from the i-esime eigenvector.

PCA_sk_ = ∑_i_^m=5^ Z_isk_ × Eg_i_/(∑_j_^n=5^ Eg_j_ (2)

In this work, PCA value ranges were proposed to establish the water quality indices for each water sample by associating the calculated value with the degree of contamination [54]. The water quality indices and the proposed stratification of PCA value ranges are as follows: (1) uncontaminated [PCA < 1.63]; (2) slightly–moderately contaminated [1.63 < PCA < 2.45]; (3) moderately–strongly contaminated [2.45 < PCA < 3.26]; (4) strongly contaminated [3.26 < PCA < 4.9]; and (5) extremely contaminated [PCA > 4.9].

The PCA value ranges were established considering the gap between the maximum and minimum values (without one outlier sample L3) and divided by three. The result of this operation is considered a base value to classify samples as uncontaminated if the PCA is below such a base value. Subsequently, the next ranges were established as 1.5, 2, and 3 times the base value.

## 4. Results and Discussion

### 4.1. As, Hg, and Tl: The Main Contaminants Identified in the Analyzed Water Bodies

The As, Hg, and Tl background values in surface water were established by considering two reference areas. The first reference area (R1) is located close to El Alamo town, and the background values for the Chiquito Stream for As, Hg, and Tl were 24.7, 33, and 21.7 µg/L, respectively. In this reference area, rainwater contacts igneous rocks (ToRP-DaP) in the Zimapan Basin, which could justify these values. The second reference area is close to the Palomas community (Figure 1e), located on the El Abra Formation. In this reference area, the water quality of four springs was characterized, and the As, Hg, and Tl concentrations exceeded the guidelines for drinking water, except for As in the El Sarro spring (see Table 2).

Table 2 shows the results of the physicochemical analyses, the concentrations of major and trace elements in the water samples, the method detection limit (MDL) for each parameter, and the guideline values (GVs) for drinking water [35,36,37]. A global chemical balance of aqueous samples allowed the estimation of a mean CI value of −9.1% with standard deviations of ±19.5% and three outliers of 95.6%, 30.7%, and 27.5% for samples corresponding to the L3, L4, and L5 leachates, respectively. In the Piper diagram, three water families could be differentiated (Figure 2). The water from two community wells (W1 and W2), the Chiquito Stream (R1), and springs located in the Palomas reference area (Table 2) were mainly bicarbonate–calcium type with a low sulfate content (4 to 20 mg/L). The river water samples (R2 to R6) were classified as a mixed type (sulfate–bicarbonate–calcium), with intermediate sulfate concentrations (118–310 mg/L). Additionally, a Piper diagram indicated that the leachates from mine waste presented high values of EC and SO_4_^2−^ and low or null concentrations of bicarbonate and carbonate (Figure 2). In addition, Pearson’s correlation for the leachates indicated that there was a strong positive correlation between Ca and SO_4_^2−^ (*R^2^* = 0.87). This suggested that gypsum could be the phase that controls the presence of SO_4_^2−^ ions in this type of sample, associated with the calculated SI (−0.6 to −2.1, cf. Section 4.5). High correlations among Al, Cu, Fe, Tl, and Zn concentrations (0.92 < *R^2^* < 0.99) evidenced the leaching of mineral sulfides contained in the ore of the abandoned La Aurora mine, which was rich in Cu-Fe-Zn-S [43,44,45]. This was also supported by the correlations observed between some metal ions (Cu, Fe, and Zn) and SO_4_^2−^ ions (*R^2^* = 0.54, 0.46, and 0.55, respectively). Blowes et al. [7] established that IOH-SO_4_^2−^ in AMWDs can release acidity when metallic ions adsorbed in these secondary minerals dissolve during runoff and then undergo hydrolysis. According to this, two hypothetical release mechanisms could be considered: (1) acid dissolution of polymetallic sulfides in AMWDs and (2) dissolution of PTE-bearing SMPs (including jarosites and IOH-SO_4_^2−^, amorphous or crystalline, or both) that migrated from flowing water to other water bodies, thus changing their SI condition [55].

### 4.2. PCA Values: A Strategy to Establish the Quality of Water Bodies

**Figure 3:**
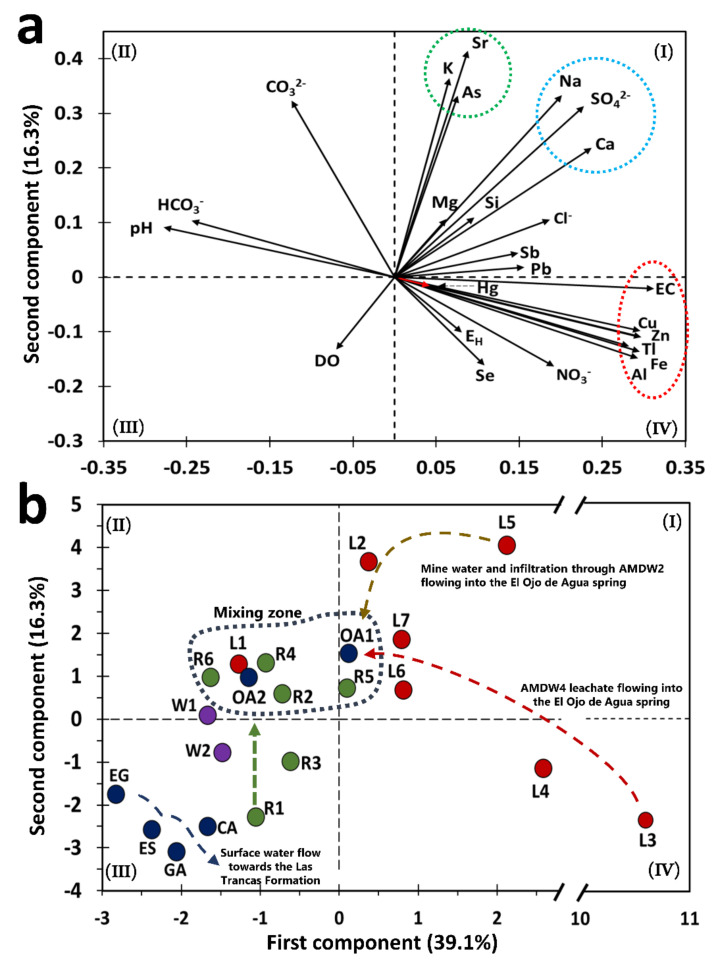
Principal component analysis for the water samples collected in the study area. (**a**) Eigenvector of the variables considered in the PCA. (**b**) Projection of the leachate samples (red circle), wells (purple circle), rivers (green circle), and springs (blue circle).

**Figure 4:**
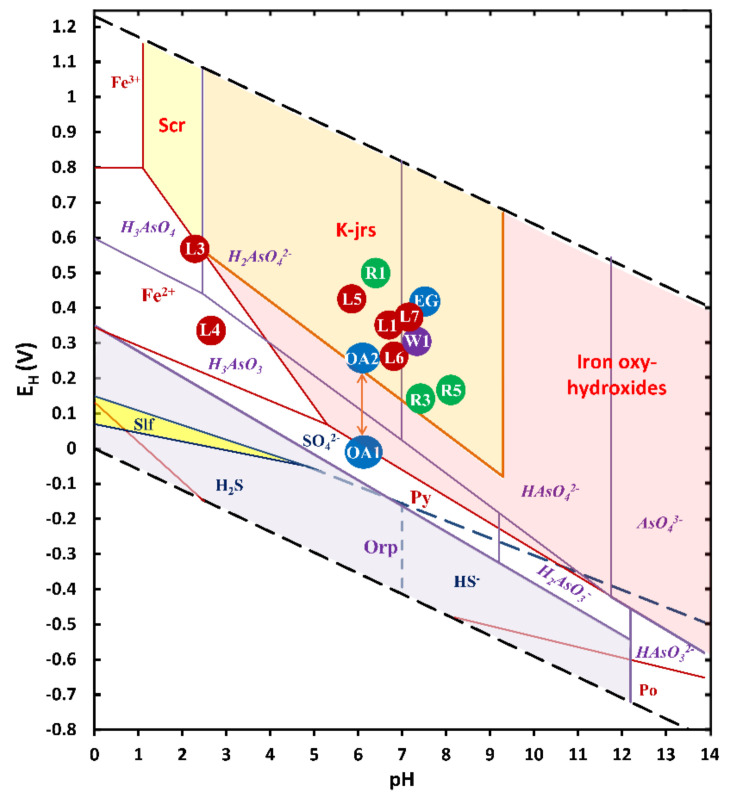
E_H_-pH diagram for the As–Fe–S system using total dissolved concentrations for [As] = 5 mM, [Fe] = 5 mM, and [SO_4_^2−^] = 0.71 mM (Constructed using MEDUSA code [62].

Principal component analysis showed a classification of variables and types of samples based on the projection of variable correlation plots that allowed the identification of four clusters (Figure 3a,b). Five principal components explained 82.2% of the total variance in the data for the analyzed water samples (Table S2). The PC1 and PC2 planes explained 55.4% of the total variance. The PC1 component was mainly composed of eigenvectors with positive values that ranged from 0.228 to 0.312, corresponding to the EC, Cu, Al, Fe, Tl, Ca, and SO_4_^2−^ variables. For the PC2 component, the eigenvectors with positive values ranging from 0.237 to 0.415 correspond to Sr, K, Na, As, CO_3_^2−^, SO_4_^2−^, and Ca variables. The main variables of PC1 suggested a chemical signature associated with weathering of polymetallic sulfide mineral phases that were originally present in the AMWD (Table S2). In addition, the main variables of PC2 suggest that the physicochemical dissolution of As-bearing secondary phases is constituted by IOH-SO_4_^2−^ (Table S2; Figure 3a). The above could explain why the presence of As in the El Ojo de Agua spring is due to the migration of fine particles of SMPs throughout the water flow until reaching the spring. The release of As in surface water derives from chemical instability associated with physicochemical conditions (Figure 4).

The eigenvectors of As, K, and Sr were projected in the same direction as the eigenvectors of SO_4_^2−^, Na, and Ca. These ions were associated with celestite, gypsum, K-jarosite, or Na-jarosite as the main SMPs carrying the elements identified in the area (Figure 3a, quadrant I). This finding was supported by both chemical and XRD analyses of stream sediments, which are described in Section 4.5 The orientation and magnitude of eigenvectors of pH and HCO_3_^−^ indicated natural water or samples from rivers not impacted by AMD (or both) (Figure 3a).

In the case of water samples, the PC1 vs. PC2 plane showed a group consisting of drinking water supply sources (springs in the Palomas reference area and wells of the La Laja community) and the Chiquito Stream (R1), which corresponds to a water sample not impacted by leachates from AMWDs (Figure 3b). These samples were characterized by neutral pH and high HCO_3_^−^ concentrations, which could be related to the limestone dissolution reaction from the El Abra Formation, flowing toward the Las Trancas Formation.

The water samples of the springs located in the Palomas reference area (GA, CA, EG, and ES) presented a range of As, Hg, and Tl concentrations between 5 and 43.2, <10 to 32.6, and 15 to 21.6 µg/L, respectively (Table 2). These concentration values were associated with the water–rock interactions between rainwater and volcanic rocks [55,56]. The highest measured As, Hg, and Tl concentrations were 43.2, 32.6, and 21.6 µg/L, respectively, in the CA spring (located at 1162 m.a.s.l.).

Although the PCA values strategy permitted classifying all water samples as a preliminary approach for the water quality index, it was necessary to deepen the analysis to find the causes that give rise to possible contamination. At this point, only four water samples were classified as uncontaminated (W1, W2, EG, and ES), three samples were classified as slightly–moderately contaminated (R1, CA, and GA), and the rest of the samples presented a grade of strongly–extremely contaminated, including the El Ojo de Agua spring (Table S3).

Figure 3b shows that the W1 and W2 samples were located outside of the surface water mixing zone. The inhabitants of the La Laja community use these wells as drinking water sources, despite this water containing 25 ± 2 µg/L As, 18 ± 10 µg/L Hg, and 16 ± 5 µg/L Tl. These values are above the guidelines for drinking water quality (Table 2); hence, it is necessary to determine the sources of these elements. Sulfate concentrations in wells were five times lower than those observed in the Santa María River or the El Ojo de Agua spring. This confirmed that W1 and W2 were not impacted by SO_4_^2−^ and could instead be associated with recent infiltration waters. The SO_4_^2−^, As, Hg, and Tl concentrations resembled the values determined in the reference zones (Table 2). The water samples observed in the mixing zone were mainly composed of river water samples (R2, R4, R5, and R6), one sample collected from the irrigation channel that surrounds AMWD1 (L1), and two samples from the El Ojo de Agua spring (OA1 and OA2).

Figure 3b shows that the W1 and W2 samples were located outside of the surface water mixing zone. The inhabitants of the La Laja community use these wells as drinking water sources, despite this water containing 25 ± 2 µg/L As, 18 ± 10 µg/L Hg, and 16 ± 5 µg/L Tl. These values are above the guidelines for drinking water quality (Table 2); hence, it is necessary to determine the sources of these elements. Sulfate concentrations in wells were five times lower than those observed in the Santa María River or the El Ojo de Agua spring. This confirmed that W1 and W2 were not impacted by SO_4_^2−^ and could instead be associated with recent infiltration waters. The SO_4_^2−^, As, Hg, and Tl concentrations resembled the values determined in the reference zones (Table 2). The water samples observed in the mixing zone were mainly composed of river water samples (R2, R4, R5, and R6), one sample collected from the irrigation channel that surrounds AMWD1 (L1), and two samples from the El Ojo de Agua spring (OA1 and OA2).

The results from L5 represent the water quality in the flooded area of the La Aurora mine (Figure 3b) from samples collected from a household water tap (L5) connected to this area. This water sample was located near AMWD2, and it was used for sanitary and cleaning services. The measured As, Hg, Tl, and SO_4_^2−^ concentrations were 94, 37.4, 21.1 µg/L, and 436 mg/L, respectively (Table 2). These elements could be considered geochemical markers of metal sulfide oxidation taking place underground [7]. In fact, L3, L4, and L5 represent water samples were severely impacted by mine waste and were classified as extremely contaminated by the PCA values (Table S3).

The L3 and L4 samples were collected from the Xichú Stream just below the AMWDs, and both samples presented high concentrations of SO_4_^2−^, Al, Cu, Fe, and Zn, which are characteristic of sites impacted by mine waste drainages [57]. The leachate samples were also characterized by high As contents as a result of water–waste interactions during infiltration through the porous spaces in tailings (L2 to L7 samples). The As, Hg, and Tl concentrations in the leachate samples ranged from 34 to 373, 8.6 to 39.7, and 10 to 918 µg/L, respectively. It is important to highlight that the samples with the highest concentrations of As, Hg, and Tl were L2, L4, and L3, respectively. Because of the SO_4_^2−^ concentrations, the L2, L4, L6, and L7 samples were considered surface waters that had been impacted by leachates from AMWDs. Figure 3b shows the confluence of these samples toward a mixing zone and the flow of leachates to the same zone. The mixing zone is constituted mainly by the Santa Maria River and El Ojo de Agua spring water samples. In the PCA diagram, L1 is a sample that surrounds a mining deposit and contains a high concentration of As (164 µg/L) and a higher concentration of DO (10.7 mg/L). This can be explained by the high photosynthetic activity observed in the field, derived from the presence of algae that promote high concentrations of DO [58,59], and low concentrations of the inorganic markers associated with mine drainage (SO_4_^2−^, Fe, Cu, and Zn).

### 4.3. El Ojo de Agua Spring: As a Potential Drinking Water Supply, Is It Impacted by Natural or Anthropogenic Events?

The El Ojo de Agua spring feeds the flow of the La Laja stream with a perennial flow rate of almost 1000 L/s. The samples collected 380 m upstream from the source of the Ojo de Agua spring (R2) and 200 m upstream of the confluence between La Laja Stream and the Santa María River (R6, Figure 1f) showed decreases in As, Hg, and Tl concentrations (Table 2). The confluence between the La Laja Stream and the Santa María River leads to equilibrium pH and E_H_ values of 7 and 397 mV, respectively (Figure 4, Table 2). These values were explained by neutralization reactions occurring because of dissolution of limestone rock and adequate aeration (see reactions in Table S4).

In the El Ojo de Agua spring, a rapid response to the variation in the E_H_ indicated an alteration of its water quality [60]. Some parameters, such as trace element concentrations of As, Pb, Zn, Sr, and Tl, also showed a response to a recent rainfall event, decreasing in concentration due to a dilution effect (Table 2). However, on a dry day, the inorganic markers associated with beudantite dissolution (Pb, As, and SO_4_^2−^) increased due to low E_H_ (10 mV) (see reactions in Table S4). Figure 4 shows different SMPs, such as scorodite, IOH, and IOH-SO_4_^2−^, their stability zones, and As-soluble species. The increase in Fe concentration (Table 2) was likely related to the entrainment of colloids or fine particles rich in SMP on rainy days [7,61].

**Figure 5:**
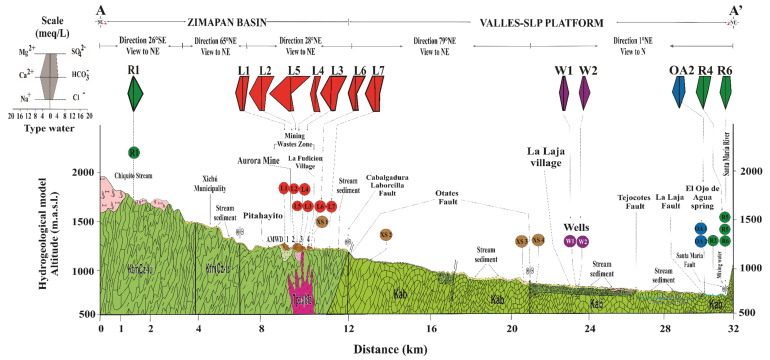
Conceptual model of water and flux of fine PTE-bearing particles in the study area. Leachate samples in red circles are under acid-neutral and oxidant conditions.

At this point, it is possible to postulate a conceptual model of water and finer PTE-bearing particle movement in the study area (Figure 5). Thus, the presence of As could be associated with the contribution of solid SMPs that carry this element from the leachate of the AMWD. It is also suggested that As could have migrated through the surface stream sediments or vadose zone until reaching this water body, as was observed in the mining district of Cerro de San Pedro in San Luis Potosí [61]. The presence of geological fractures filled with iron oxides and carbonates has been documented in a transect transverse to the Xichú Stream channel, which crosses the area of mine waste dumps [32]. These interstitial iron oxides could be associated with secondary mineral products of the pyrite oxidation present in the AMWDs and the natural oxidation of the sulfides in rocks. The iron oxides that migrate from waste dumps as fine materials are potential As-bearing SMPs. In these phases, As can be occluded, coprecipitated, or adsorbed. Arsenic that is adsorbed or coprecipitated on IOH particles (amorphous or crystalline, or both), which fill the fractures in the area, can be released by ion exchange reactions or by reductive dissolution when the particles migrate to sites with anoxic conditions [55].

Additionally, the water quality response from the El Ojo de Agua spring could be associated with the effect of geological water–rock interactions between the Zimapan Basin, the Valle-San Luis Potosí Platform, and the Las Trancas Formation. This geological condition has led to an interesting finding that explains the presence of Hg in the springs of the Palomas reference zone and the El Ojo de Agua spring. The Hg concentrations can be attributed to rainwater–mineralized igneous rock interactions (ToRP-DaP). In fact, near this zone, under similar geological conditions, cinnabar ores occur, and low quantities of Hg are present in the volcanic rocks [63]. It is important to notice that there are not historical records or physical evidence of amalgamation processes carried out in this site, although there is evidence of an ancient mercury mine at 70 Km SE of the site. For instance, Martínez-Trinidad et al. [64] reported concentrations of 0.6 to 687 mg/kg Hg in sediments near San Joaquin Querétaro. 

### 4.4. Isotopic Analysis of δ^34^S_sulfate_, δ^18^O_sulfate_, and Sulfate Presence in the El Ojo de Agua Spring

**Figure 6:**
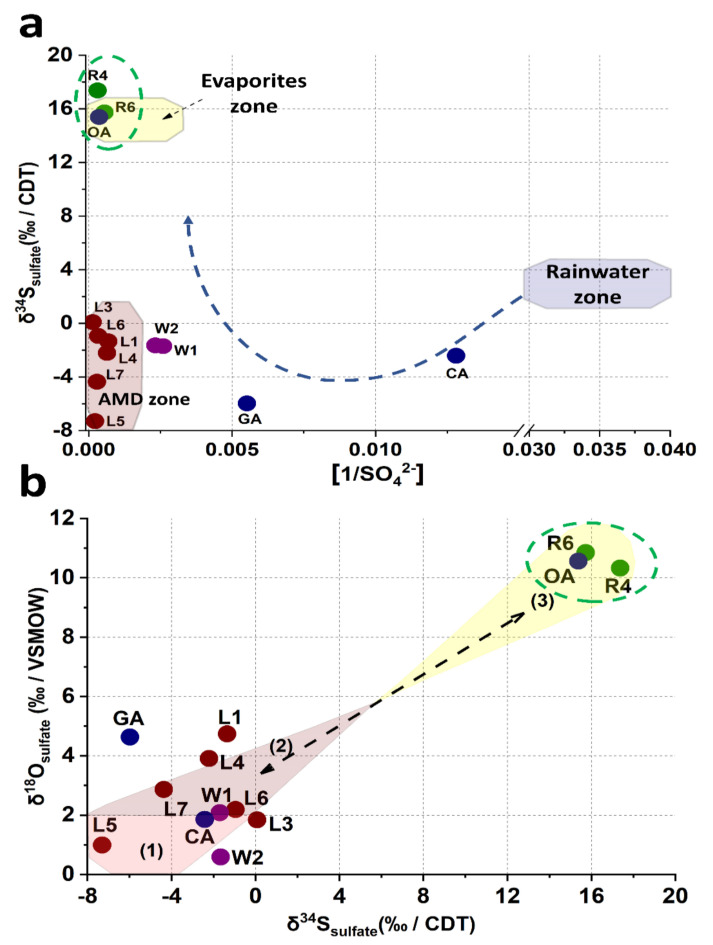
Isotopic analysis. (**a**) δ^34^S_sulfate_ values vs. the 1/[SO_4_^2−^] ratio showing the rainwater zone and evaporite zones and defining a new zone in this study for water quality associated with AMD produced by sulfide oxidation.(**b**) Isotope values of δ^18^O_sulfate_ and δ^34^S_sulfate_ associated with each input and output flux of sulfate. The shadow areas represent the identified processes that correspond to (1) mineral sulfide oxidation, (2) precipitation of sulfate secondary minerals by oversaturation, and (3) old Cretaceous evaporite dissolution from the El Abra Formation.

The origin of sulfate in the El Ojo de Agua spring was determined by the isotopic composition of δ^34^S_sulfate_ and δ^18^O_sulfate_. Several authors have established low or negative isotopic compositions of δ^34^S_sulfate_ and δ^18^O_sulfate_ associated with sulfide oxidation in mining areas [65,66,67,68]. In Figure 6a, samples L1, L3, and L7 are grouped in a cluster with similar δ^34^S_sulfate_ vs. 1/[SO_4_^2−^] ratios, located in a region characterized by sulfide oxidation, which in this study is labeled the AMD zone. These results supplement a similar study by Brenot et al. [65]. The well water samples (W1 and W2) from the La Laja community had a δ^34^S_sulfate_ vs. 1/[SO_4_^2−^] ratio closer to the ratio (δ^34^S_sulfate_ vs. 1/[SO_4_^2−^]) of leachate samples but were also influenced by surface runoff and infiltration of rainwater connected through the multiple geological faults, documented in the Palomas reference zone [38]. The δ^34^S_sulfate_ vs 1/[SO_4_^2−^] ratio, attributed to the GA and CA springs, is mainly associated with rainwater values reported by Brenot et al. [65]. Finally, a third cluster, attributed to the evaporite zone by Brenot et al. [65], was identified and conformed to that of the El Ojo de Agua spring, the Santa María River (R4), and its confluence with the La Laja Stream (R6), which is different from the two previously identified clusters (Figure 6a). Previous results established that the presence of the SO_4_^2−^ ions in the El Ojo de Agua spring came from a different source than mining residues in the study area, suggesting that there was no direct connection between the sulfates generated by the mine wastes and sulfate ions found in this spring. The isotopic compositions of δ^18^O_sulfate_ and δ^34^S_sulfate_ are projected in Figure 6b. Isotopes values of δ^18^O_sulfate_ could be associated with three different processes of input and output SO_4_^2−^ fluxes identified in the study zone, which are associated with the following: (1) mineral sulfide oxidation weathering by atmospheric oxygen (L5 and L7), with a range of values of δ^18^O_sulfate_ between +1 and +1.8; (2) SO_4_^2−^-bearing secondary mineral precipitation by oversaturation of SO_4_^2−^ via AMWDs weathering, with a range of values of δ^18^O_sulfate_ between +2.2 and +3.9; (3) the dissolution of old Cretaceous evaporites of SO_4_^2−^, with a range of values of δ^18^O_sulfate_ between ~+10 and +11 [68,69]. These values agree with those reported by Otero et al. [68] and Claypool et al. [69].

The isotopic signature in the El Ojo de Agua spring is the same as that in the Santa Maria River, and this last isotopic signature agrees well with the δ^18^O_sulfate_ and δ^34^S_sulfate_ isotopic signatures in Cretaceous evaporites for marine evaporite SO_4_^2−^ dissolution reported by Claypool et al. [69]. Basáñez-Loyola et al. [70] described that the El Abra Formation was constituted by three depositional environmental events, among which, two were associated with evaporitic lagoons, which is in line with the isotopic results observed in this study. Therefore, it is possible to postulate that the sulfate incorporated into the El Ojo de Agua spring originated from the rock–water interactions of the geological formation, within which it was structurally confined via the evaporitic SO_4_^2−^ content in the El Abra Formation. Otherwise, the origin of SO_4_^2−^ did not correlate with the origin of As in the El Ojo de Agua spring because the increased contribution of sulfates could be related to an underground geological structure connecting the Santa María River and the El Ojo de Agua spring (Figure 5) via the Santa María and La Laja lateral faults (Figure 1b). Likewise, the concentrations of SO_4_^2−^ in the samples from the Santa María River and isotopic compositions of δ^34^S_sulfate_ and δ^18^O_sulfate_ remained relatively constant, as was observed by Brenot et al. [64] regarding the temporal and spatial variations in water quality in the Moselle River Basin. Thus, while the presence of SO_4_^2−^ could be of natural origin, the presence of As could not.

### 4.5. Arsenic Content in the El Ojo de Agua Spring and its Relationship with the Dragging of Fine Particles via Hydric Erosion

**Figure 7:**
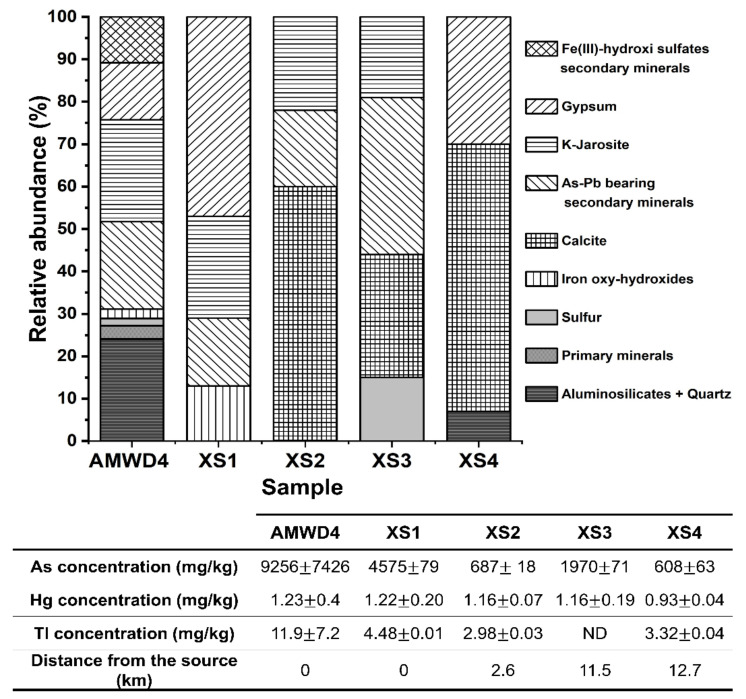
Relative abundance of mineral phases identified by XRD in the wall of AMWD4, stream sediment samples, and the corresponding As, Hg, and Tl total concentrations (mg/kg). The As–Pb-bearing secondary minerals are represented by beudantite, Pb-jarosites, Lautite, Lanarkite, Segnetite, scorodite, and Sarkinite. Fe(III)-hydroxi-sulfate secondary minerals correspond to ferricopiapite and coquimbite. Iron oxides correspond to goethite and lepidocrocite. Primary minerals correspond to pyrite and chalcocite (see Table 3).

**Figure 8:**
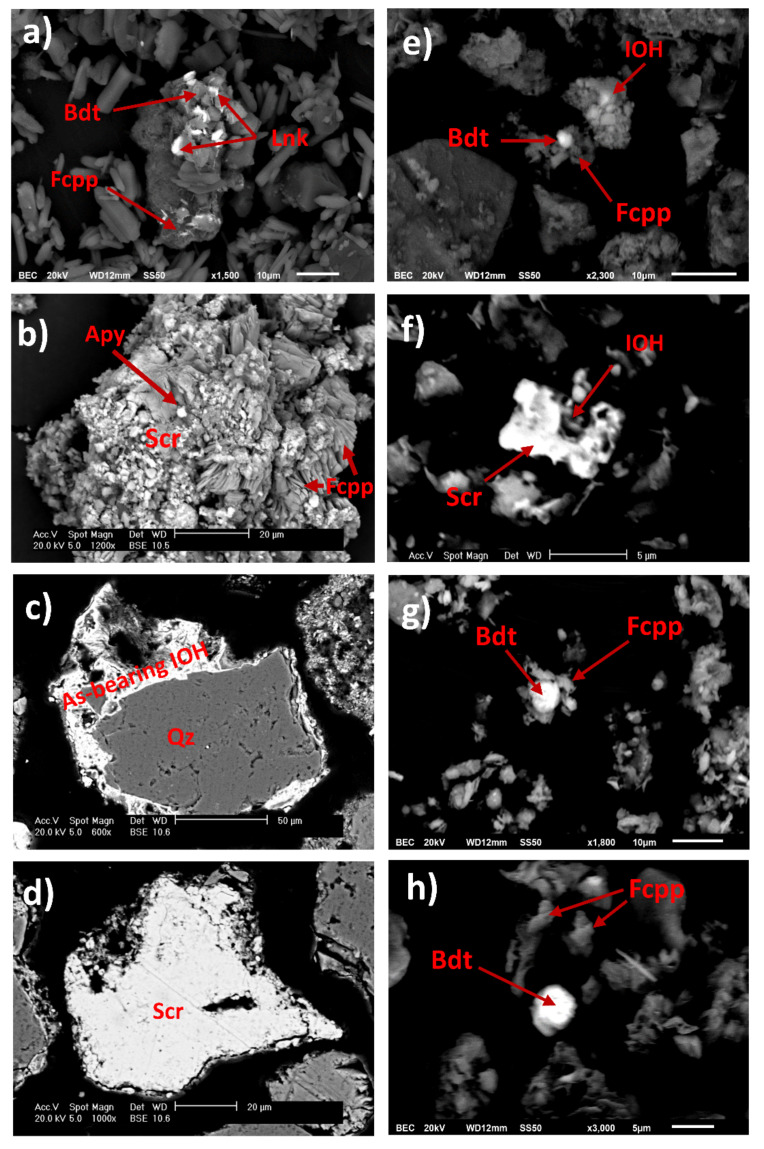
Photomicrographs of particles present on the wall of AMWD4 (**a**) and (**b**); stream sediments XS1 (**c**) and (**d**), XS2 (**e**), XS3 (**f**) and (**g**), and XS4 (**h**). SEM-EDS point analysis suggests the presence of arsenopyrite (Apy), beudantite (Bdt), scorodite (Scr), As-bearing IOH, ferricopiapite (Fcpp), and lanarkite (Lnk) (see Table S5).

XRD, XRF, and SEM analyses were paramount in elucidating the origin of As in the El Ojo de Agua spring and its relationship with the contributions of secondary minerals from mine waste dumps (Figure 7 and Figure 8). SMPs identified by XRD analysis were associated with the weathering products of AMWDs from the reactions described in Table S4. In fact, As-bearing SMPs could contain As that was coprecipitated (in gypsum or jarosites), adsorbed (in jarosites or goethite), or even part of the SMP structure (e.g., scorodite, beudantite, or IOH phases–amorphous or crystalline). In this study, the AMWDs were considered the sources of As due to an average concentration of 9300 ± 7400 mg/kg, which shows a heterogenic distribution and great diversity of primary and As–Pb-bearing SMPs (Figure 7).

The SEM observations show that fine particles such as lanarkite and beudantite were disseminated and scattered around ferricopiapite in wall lateral AMWD samples (Figure 8a). In addition, it was even possible to observe Scorodite particles, one of the most representative As-bearing mineral phases in AMWD (Figure 8b,d,f). Scorodite mineral particles were evidenced in the walls of AMWD4, probably due to arsenopyrite oxidation and gradual conversion into Fe arsenate via AsO_4_^3−^ into scorodite in AMD conditions as it has also been observed by Dill et al. [71] (Figure 8b and Figure 4).

To trace the fine particle dispersion, sediment samples taken near and far from AMWDs were also observed. The sediments collected near AMWD4 (XS1) had an As concentration of 4575 ± 79 mg/kg, and XRD results established that these sediments were mainly composed of quartz and aluminosilicates (58% of the bulk). However, the results indicated that the remaining 42% was composed of gypsum (47%), K-jarosite (24%), beudantite (16%) (an As–Pb-bearing secondary mineral), and goethite (13%). The presence of As-bearing IOH was observed in the SEM microphotographs of the XS1 stream sediment (Figure 8c) and was likely related to precipitation or coprecipitation processes (or both) [41,55,72]. In addition, in XS1, it is possible to observe the drag of SMPs from AMWD4 to sediment streams through the physical erosion of fine particles (~80 µm) of scorodite (Figure 8d).

The XS2 stream sediment located 2.6 km downstream from AMWD4 contained 690 ± 20 mg/kg As. The decrease in As concentration indicated a dilution process with respect to sample XS1. The XS2 stream sediments were mainly composed of the mineralogical phases of quartz and aluminosilicates (76% of the bulk). According to XRD results, it was possible to verify that the remaining 24% of the bulk consisted of carbonate (60%), Jrs (22%), and sarkinite (18%) phases. At this site, the presence of fine particles (<10 µm) of beudantite, ferricopiapite, and IOH (Figure 8e) could explain the migration of As from the dumps of mining residues by hydraulic flow [73].

The XS3 stream sediment, located 11.5 km downstream from AMWD4, is a fine particle accumulation zone. This sample presented an As concentration of 1970 ± 71 mg/kg, and the XRD results indicated the presence of As-bearing SMPs, such as beudantite and K-jarosite, produced by AMD (Figure 7). In addition, the SEM micrographs of this sample showed fine beudantite particles (<10 µm) occluded in ferricopiapite, scorodite, and IOH mineral phases (Figure 8f,g). However, it is shown that, probably after hydric erosion processes, As-bearing fine particles (beudantite) are released, as they are no longer surrounded by ferricopiapite (Figure 8g,h). This fragmentation could be a consequence of either hydric erosion or abrasion in the accumulation zone. The presence of As-bearing fine particles in XS3 and XS4 could be attributed to migration in aqueous suspension or promoted by dragging due to the hydraulic flow. The XS4 sediment sample presented a predominance of Qz, K-Fe aluminosilicates, and carbonates, as well as a dilution in the As concentration up to 610 ± 60 mg/kg (Figure 7), with respect to XS3. This last value could be associated with the dilution effect on the sediments of the Xichú Stream due to the incorporation of geological materials carried by the Guamuchil Stream. The XRD results for the stream sediments showed no evidence of the high concentrations of Tl and Hg; however, the impact of these elements via the AMWDs should be further studied (Figure 3a and Figure 7).

Considering the formation of SMPs and their possible As migration from the AMWDs, the SI of the mineral phases of interest were calculated (Table 3). The SI for each aqueous samples showed a sequence of chemical stability as a function of their chemical and physicochemical conditions.

The presence of subsaturated secondary minerals was observed in the samples collected near the AMWD of the La Aurora mine (L1–L7), indicating that the minerals in these samples were under dissolution conditions and could have been supplying As that was adsorbed or coprecipitated, or both. It was determined that the L5, L2, L6, and L7 samples were oversaturated in goethite and lepidocrocite. The precipitation of these minerals would have controlled the mobility of As in the mine waste area (Figure S1). Asta et al. [72] determined that the concentration of As in areas contaminated by AMD was naturally attenuated by the formation of new mineral phases such as schwertmannite, K-Jrs and goethite, which play an important role in the removal of As through adsorption and coprecipitation processes. Fine particles of iron oxide, as well as IOH-SO_4_^2−^ precipitates, are dispersed downstream away from mine waste dumps by water flow. The range of SI values calculated for each aqueous sample (Table 3) supports the hypothesis that when As-bearing mineral phases (such as scorodite, beudantite, coquimbite, ferricopiapite, K-jarosite, adamite, segnitite, and guerinite) reach the El Ojo de Agua spring by hydric erosion, As is released via a mineral dissolution mechanism (Figure S2).

**Table 3:** Mineral phases present in the samples from the study area.

Abbreviation ^1^	Name	Chemical Formula	Solid Sample		Index Saturation Range ^2^
Mineral Gangue			AMWD	Sediments	
Qz	Quartz	SiO_2_	√	√	
Als	Aluminosilicate	K (AlSi_3_O_8_)	√	√	
Cal	Calcite	CaCO_3_		√	−0.8 to −1.2
Primary phases					
L	Lautite	CuAsS	√	√	(*)
Ccp	Chalcopyrite	CuFeS_2_		√	(*)
Cct	Calcocite	Cu_2_S	√		(*)
Sp	Sphalerite	ZnS	Hypothetic mineral phase		(*)
Gtn	Gratonite	PbS_2_As_2_S_3_	√		(*)
Py	Pyrite	FeS_2_	√		(*)
Rlg	Realgar	As_4_S_4_	√	√	(*)
Orp	Orpiment	As_2_S_3_	√		(*)
Slf	Sulfur	S	√	√	(*)
Iron oxy-hydroxides					
He	Hematite	Fe_1.8_H_0.66_O_3_	√	√	4.1 to −17.6
Gth	Goethite	Fe_2_O_3_ · H_2_O	√	√	1.6 to −9.2
Lpd	Lepidocrocite	Fe^+3^O(OH)	√		2.8 to −10.7
Fhy	Ferrihydrite	Fe_2_(OH)_3_ · 0.5H_2_O	Hypothetic mineral phase		1.3 to -12.3
Secondary mineral phases					
Gy	Gypsum	CaSO_4_ · 2H_2_O	√	√	−0.6 to −2.1
Scr	Scorodite	FeAsO_4_ · 2H_2_O	√	√	−1.4 to −17.7
Bdt	Beudantite	Pb(Fe_2.5_Al_0.46_)(As_1.07_O_4_)SO_4_(OH)_6_	√	√	−0.2 to −44.2
Pb-Jrs	Plumbojarosite	(Pb_0.43_K_0.14_)Fe_3_(SO_4_)_2_(OH)_6_	√	√	
K-Jrs	K-Jarosite	(K_0.86_(H_3_O)_0.14_)Fe_3_(SO_4_)_2_(OH)_6_	√	√	−5 to 30.7
Adm	Adamite	Zn_2_(AsO_4_)(OH)	√		−5.5 to −22.9
Lnk	Lanarkite	Pb_2_(SO_4_)O	√		−8.7 to −16.6
Bch	Brochantite	Cu_4_(SO_4_)(OH)_6_	√		−6.9 to −30.1
Cld	Claudetite	As_2_O_3_	√		−10.5 to −27.4
Sgn	Segnetite	PbFe_3_(AsO_4_)(AsO_3_OH)(OH)_6_	√		−12.2 to −45.2
Cqm	Coquimbite	Fe_1.54_Al_0.46_(SO_4_)_3_ · 9H_2_O	√		−28.1 to −35.1
Grn	Guerinite	Ca_5_(AsO_4_)_2_(AsO_3_(OH))(H_2_O)_4_	√	√	−17.8 to −39.3
Skn	Sarkinite	Mn_2_AsO_4_(OH)	√	√	−26.5 to −47.8
Fcpp	Ferricopiapite	Fe_4.67_(SO_4_)_6_ (OH)_2_ · 20H_2_O	√		−34 to −46

^1^—Mineral abbreviation was assigned following Whitney and Evans [74]. ^2^—Index saturation range was determined with PHREEQC, using the values in Table 2 for each aqueous sample. √—Mineral phase identified in AMWD and sediments by XRD. (*)—Mineral phase not modeled because hydrogen sulfide concentrations in solution were not determined.

## 5. Conclusions

In this study, the qualities of different bodies of water in the XMD were determined, and among other pollutants, the presence of As, Hg, and Tl was measured, respectively. Based on the chemical and physicochemical parameters measured and, mainly through the PCA statistical tool, a water quality classification of the different collected samples was proposed, resulting in four water samples classified as uncontaminated (W1, W2, EG, and ES), three as slightly–moderately contaminated (R1, CA, and GA), and the rest of the samples classified as highly–extremely contaminated, including a potential source of drinking water in the region, i.e., the El Ojo de Agua spring.

Additionally, the isotopic compositions of δ^18^O_sulfate_ and δ^34^S_sulfate_ allowed the identification of three processes of sulfate fluxes (input and output fluxes) in the study area that correspond to (1) the oxidation of mineral sulfides; (2) the precipitation of secondary sulfate mineral phases produced by AMWD weathering; and (3) sulfate dissolution from evaporites contained in the El Abra Formation. In fact, the isotopic signature of δ^18^O_sulfate_ and δ^34^S_sulfate_ made it possible to establish that the origin of sulfates in the Santa Maria River and the El Ojo de Agua spring is similar and is associated with the dissolution of evaporites.

PCA and Pearson correlations made it possible to establish that the main source of As contamination in the study area is related to the weathering products of AMWDs. These tailings deposits, without coverings, are exposed to environmental conditions, generating AMD that percolates through tailings, promoting the formation of new As-bearing secondary phases. Hence, the impact of the tailings on the water bodies was associated with the physical migration promoted by the hydraulic drag of these secondary phases. The secondary phases, in the form of fragmented particles, decrease in size while they are dragged, either by erosion or by abrasion processes (or by both) during their migration. They also undergo fracturing processes and release fine particles with high arsenic content. This was evidenced through chemical analyses performed with scanning electron microscopy–energy dispersive X-ray spectrometry (SEM–EDS) on sediment samples collected along the stream channels that flow into the El Ojo de Agua spring. It was not possible to identify any fine particle carriers of Tl and Hg, so it is assumed that the presence of these elements is related to other processes that favor their entry to the spring. From the PCA analyses and Pearson’s correlations, it is hypothesized that the fraction of thallium present in the El Ojo de Agua spring could be related to the weathering processes of AMWDs. However, the presence of Tl and Hg in the springs in the Palomas reference zone suggests that an important contribution to consider could be the dissolution of these elements from igneous rocks in the study area when rainwater interacts with them and then percolates through the limestone of the El Abra Formation, which, however, needs further review.

Finally, the results suggest that the presence of sulfates in the spring is of natural origin—the presence of As is related to the dissolution of fine particles of the secondary phases (resulting from precipitation from mine waste leachates) that carry this element when they enter the body of water, and it is not yet possible to establish reliably whether, in the El Ojo de Agua spring, the presence of Tl and Hg is of natural or anthropogenic origin.

Water contamination by Tl and Hg is evidenced in this work only as a preliminary finding. It is necessary to carry out in-depth studies that allow us to reliably explain their presence; however, the authors of this work consider it important to highlight this result, due to public interest in the impacts that these elements could have on vulnerable communities in the region. This is important when the geographical location is difficult to access and the infrastructure to acquire other water resources for human consumption is lacking.

## Figures and Tables

**Table 1:** Geographical location, type, and physical condition of the aqueous and solid samples collected in the study area.

Label Sample	UTM Coordinates		Altitude (m.a.s.l.)	Type of Sample	Description of the Sampling Site
	X	Y			
L1	392,742	2,358,724	1120	Leachates	Water sample from handmade rainwater channel for local irrigation, located on the lateral wall of AMWD1.
L2	392,922	2,359,056	1104	Leachates	Leachate water sample produced by water infiltration through the AMWD2.
L3	392,958	2,359,158	1118	Leachates	Water sample collected from the Xichú Stream, impacted by the runoff of leachate water from the walls of AMWD4.
L4	392,972	2,359,161	1083	Leachates	Water sample collected from the Xichú Stream, impacted by the runoff of water leachate from the lateral walls of AMWD2.
L5	393,062	2,359,130	1140	Leachates	Mine water sample collected from the mineralized zone of La Aurora, located in the La Fundición community.
L6	393,081	2,359,369	1100	Leachates	Water sample from handmade rainwater channel for local irrigation, located on the lateral wall of AMWD4.
L7	393,483	2,360,138	1041	Leachates	Water sample from the Xichú Stream, impacted by leachate runoff located 850 m downstream from AMWD4.
W1	403,068	2,365,735	846	Water well	Water sample from drinking water well of the La Laja community.
W2	413,488	2,365,725	849	Water well	Water sample from handmade drinking water well of the La Laja community, at 100 m from W1 sample.
R1	386,596	2,355,721	1610	Streams	Water sample from the Chiquito Stream located close to El Alamo community at 1634 m.a.s.l. at 8.5 km upstream from AMWD1.
R2	402,933	2,372,130	761	Streams	Water sample collected on the La Laja stream, located at 380 m downstream from the El Ojo de Agua spring.
R3	402,846	2,372,215	765	Streams	Water sample from the Santa María River, located 100 m before its confluence with the La Laja Stream.
R4	402,614	2,372,157	763	Streams	Water sample from the Santa María River, located 250 m before its confluence with the La Laja stream.
R5	402,929	2,372,254	754	Streams	Water sample from the Santa María River, located 100 m after its confluence with the La Laja stream.
R6	403,063	2,372,233	759	Streams	Water sample from the Santa María River, located 200 m after its confluence with the La Laja stream.
OA	402,758	2,371,729	756	Spring	Water sample from the El Ojo de Agua Spring on the La Laja Stream bed.
GA	413,520	2,365,858	1029	Spring	Water sample from Guayaba Agria spring.
CA	413,539	2,365,666	1163	Spring	Water sample from El Carricillito spring.
EG	414,872	2,364,717	1436	Spring	Water sample from El Gato spring.
ES	415,394	2,364,923	1550	Spring	Water sample from El Sarro spring.
AMWD4	393,085	2,359,291	1110	Waste	Solid samples of wastes taken from AMWD4.
XS1	393,106	2,359,266	1103	Sediment	Stream sediment from Xichú River impacted by fine particles generated by mineral secondary phases, precipitated chemically on the walls of the mining waste deposit. Sample located in the lower part of the AMWD4.
XS2	394,683	2,360,714	1040	Sediment	Surface layer of sediments from the Xichú stream in the low energy zone, 2.6 km downstream from the AMWD4.
XS3	402,605	2,362,269	895	Sediment	Superficial layer of stream sediments from Xichú Stream in the zone of low energy and produced by its drying, before its confluence with the Guamuchil stream.
XS4	403,015	2,362,875	860	Sediment	Superficial layer of stream sediments from Xichú Stream in the zone of low energy and produced by its drying, after its confluence with the Guamuchil stream.

**Table 2:** Physicochemical parameters and chemical concentrations of cations, anions, and trace elements in different types of aqueous samples collected in the study area.

Variable	Units	Aqueous Samples																						
		Leachates							Streams						Wells		Springs							
		L1	L2	L3	L4	L5	L6	L7	R1	R2	R3	R4	R5	R6	W1	W2	OA1	OA2	GA	CA	EG	ES	MDL	GV
T	(°C)	31.2	28.6	19.9	21.3	44.9	21.5	26.4	27.2	27	31.5	32.3	28.6	27.9	24.9	24.7	27.9	27.2	24.5	20.9	17.9	17.7	−	-
pH	-	6.6	6.1	1.8	2.6	5.9	6.7	6.9	6.2	6.3	7.2	6.7	7.6	7	6.5	6.6	6	6.2	7.1	6.8	6.9	6.9	−	6.5 to 8.5 [36]
E_H_	mV	355	382	606	380	416	239	355	406	402	193	377	215	397	343	396	10	359	379	349	362	374	−	-
DO	mg/L	10.7	4.3	3.5	6.4	0.5	3.1	6.3	4	2.8	4.1	6.4	2.3	4	5.1	3.2	3.2	4.2	5.2	4.2	4.8	6	−	-
EC	μS/cm	697	1,234	8,480	2,868	1,954	691	1,102	810	829	1,016	1,008	970	840	436	466	967	825	600	668	466	456	−	-
Ca	mg/L	73.8	169.6	314.4	69.2	205.6	86.3	157.6	27.4	110.5	53.3	111.5	113.5	88.5	63.2	67.1	113	112	30.4	76.4	73.6	72.8	0.03	-
Mg		17.6	28.7	33.7	11.3	40.9	10.1	26.9	5.6	23.8	15.2	35.3	27.7	25.3	9.2	9.8	27.5	24.6	37.4	37.3	18.1	25.2	0.02	-
Na		22.6	29.6	31	21.8	42.3	20.1	29.6	6.6	6.8	4.5	8.5	11	7.1	8.2	8.3	10.6	7.1	0.4	0.9	<0.02	<0.02	0.02	200 [36]
K		5.1	6.4	2.6	6.8	22.2	7.2	5.3	1.7	2	1.5	7.1	2.7	4.6	12.3	3.3	2.5	3.1	1	0.9	1.8	1.3	0.12	-
Cl^−^		21.6	80.7	92.2	75	30.5	68.1	116	<0.2	<0.2	50.5	<0.2	55.6	<0.2	20.7	<0.2	14	<0.2	19.4	18.7	<0.2	<0.2	0.2	250 [36]
NO_3_^−^		<10	<10	98.2	53.1	11.4	10.1	31	75	<10	13	<10	<10	<10	<10	18.7	17.6	<10	42	<10	<10	<10	10	50 [35] 44.2 [36]
HCO_3_^−^		126.4	206.4	<10	<10	172.9	57.6	215.8	110.2	248.4	61.4	215.7	229.2	270.5	233.6	282.2	265.4	263.7	206.4	250.1	350.5	274.2	10	-
CO_3_^2−^		15.5	18.1	<10	<10	18.1	22.8	12.9	12.9	20.6	10.7	15.5	12.1	12.9	10.3	7.7	15	18.1	5.2	2.6	10.3	15.5	10	-
SO_4_^2−^		140	415	686	150	436	287	327	4	139.9	118.4	310.6	180.5	175.8	37.4	41.3	206.7	164.1	17.4	7.5	14.6	20	0.2	400 [36]
Si^Tot^		18.9	16.7	29.5	8.4	18.9	6.9	15.3	23.3	32.4	3.7	20.3	8.4	17	20.5	21.2	8.5	16.2	9.6	8.1	9.1	7.6	0.02	-
As	μg/L	164	373	69.3	34.4	93.6	47	89	24.7	45	17.5	14.7	44.8	47.2	26.9	23.2	121	81.5	28.2	43.2	9.1	5	3	10 [36] 25 [36]
Hg		18.1	19.6	18	39.7	37.4	<10	33	33	23.4	17.7	36.9	<10	<10	<10	24.6	<10	33	22.2	32.6	22.3	10.1	10	6 [35] 1 [36]
Tl		14.5	<10.3	918	16.5	21.1	73	22.3	21.7	22.7	18.7	30.4	26.8	16.7	12.4	19.3	49.1	23.6	15	21.6	17.1	18.7	10	13 [37]
Pb		<5	<5	27.4	6.9	<5	6.9	9.5	<5	<5	5.7	<5	27.5	<5	8.3	5.2	55.2	<5	<5	<5	<5	<5	5	10 [35,36]
Fe		<6.2	6.2	1, 214	100	11	33.6	<6.2	33.7	43	10.4	<6.2	16.4	<6.2	<6.2	8.2	<6.2	17.9	<6.2	7.65	<6.2	<6.2	6.2	300 [36]
Cu		<11.9	<11.9	2, 559	25.1	35.5	16.2	<11.9	<11.9	<11.9	<11.9	<11.9	<11.9	<11.9	6.1	6.6	11.9	<11.9	<11.9	<11.9	<11.9	<11.9	11.9	2000 [35]
Zn		<5	22.4	12,924	52	314	11.5	<5	5.3	<5	14.7	<5	46.4	<5	<5	8.5	12.2	<5	8.4	<5	<5	<5	5	3000 [35]; 5000 [36]
Sr		579	1, 054	436	1, 107	2, 042	380	1, 105	191	924	602	1, 220	656	949	294	312	1, 211	958	67	65	95	62	3.4	4000 [35]
Al		5.6	11.4	16, 356	303	23.6	24.7	<5	10.7	12.8	7.8	<5	36.1	<5	<5	<5	14.8	5.2	13	26.3	<5	<5	5	200 [36]
Sb		15.6	16.2	34.2	64	21.2	69.2	20.1	17.7	16.4	28.4	15.6	57.3	14.6	13	15.8	43	13.4	11.8	15.4	12.2	11.4	9.7	20 [35]
Se		<7.3	<7.3	14	15.4	10.2	14.6	11.3	11	8.32	14	<7.3	15.3	<7.3	<7.3	<7.3	14.2	<7.3	16.5	<7.3	<7.3	21.6	7.3	40 [35]
CI	%	12.4	−18	95.6	30.7	27.5	−42	−23.8	−33.3	13	−22.6	−12.1	−7.3	−17.3	−12.8	−23.8	−11.6	4.1	−7.1	37.5	15.4	16	−	-

E_H_—redox potential, relative to a standard hydrogen electrode (mV); CI (%)—charge imbalance [50]. CI mean was –9.1% with standard deviations of ± 19.5% and three outliers of 95%, 30.7%, and 25.5%; MDL—method detection limit; GV—guideline values for drinking water, established by the World Health Organization [35], the Mexican normative [36], or the Environmental protection agency [37].

## Data Availability

All supporting data have been included in this study and are available from the corresponding authors upon request.

## Supplementary Materials

The following are available online at https://www.mdpi.com/article/10.3390/toxics9110307/s1, Figure S1: Climatograph of the study area based on the average of monthly data of the period 1951–2015 [30], Figure S2: Saturation index estimation of the main mineral phases identified by XRD in samples from AMDW4 when they are in contact with the physicochemical quality of leachate, river, well, and spring samples using PHREEQCI (2.15 software), Table S1: Pearson’s correlation coefficient between the physicochemical parameters and ions quantified in the leachates, river, and spring water samples in the study area, Table S2: Eigenvectors from principal component analysis for the different types of water samples collected in the study area (with transformed variables to square root values), Table S3: Water quality classification considering the PCA values calculated with physicochemical parameters and chemical compositions of aqueous solution samples, Table S4: Proposed reactions involved in the oxidation of primary sulfide minerals and precipitation or dissolution reactions that produce the SMPs that were identified by XRD in the waste and sediment samples collected from AMDW4 [7,14,51], Table S5: SEM–EDS analysis of waste and stream sediment samples: AMWD (a,b), XS1 (c,d), XS2 (e), XS3 (f,g), and XS4 (h). SMPs were assigned by mass balance reconstruction based on chemical composition.
