# Supplementary material for: Presence of Arsenic in Potential Sources of Drinking Water Supply Located in a Mineralized and Mined Area of the Sierra Madre Oriental in Mexico
Source: Toxics. 2021 Nov 15;9(11):307. doi: 10.3390/toxics9110307 (PMC8618315; doi:10.3390/toxics9110307)
Supplement: Supplementary file 1 [file toxics-09-00307-s001.zip › toxics-1444185-sm-final.pdf]

toxics MDPI

Supplementary Materials: Presence of Arsenic in Potential Sources of Drinking Water Supply Located in a Mineralized and Mined Area of the Sierra Madre Oriental in Mexico

Victor Manuel Escot-Espinoza, Yann Rene Ramos-Arroyo, Isabel Lázaro, Isidro Montes-Avila, Leticia Carrizalez-Yañez and Roberto Briones-Gallardo

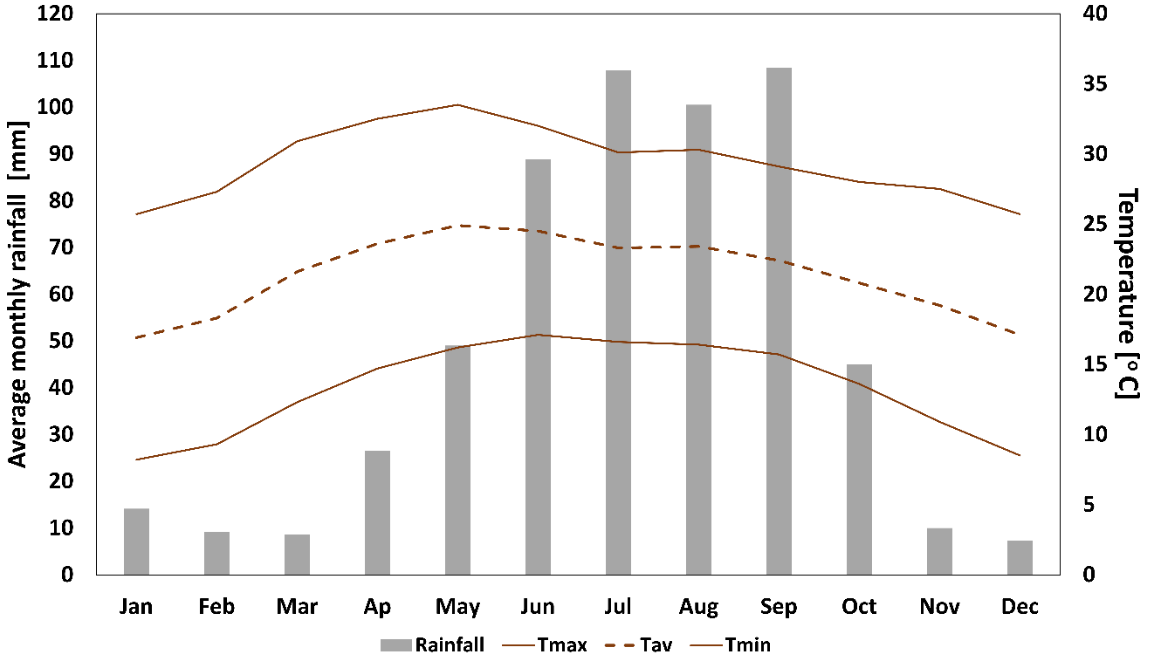

Figure S1. Climatograph of the study area based on the average of monthly data of the period 1951 to 2015 [30].

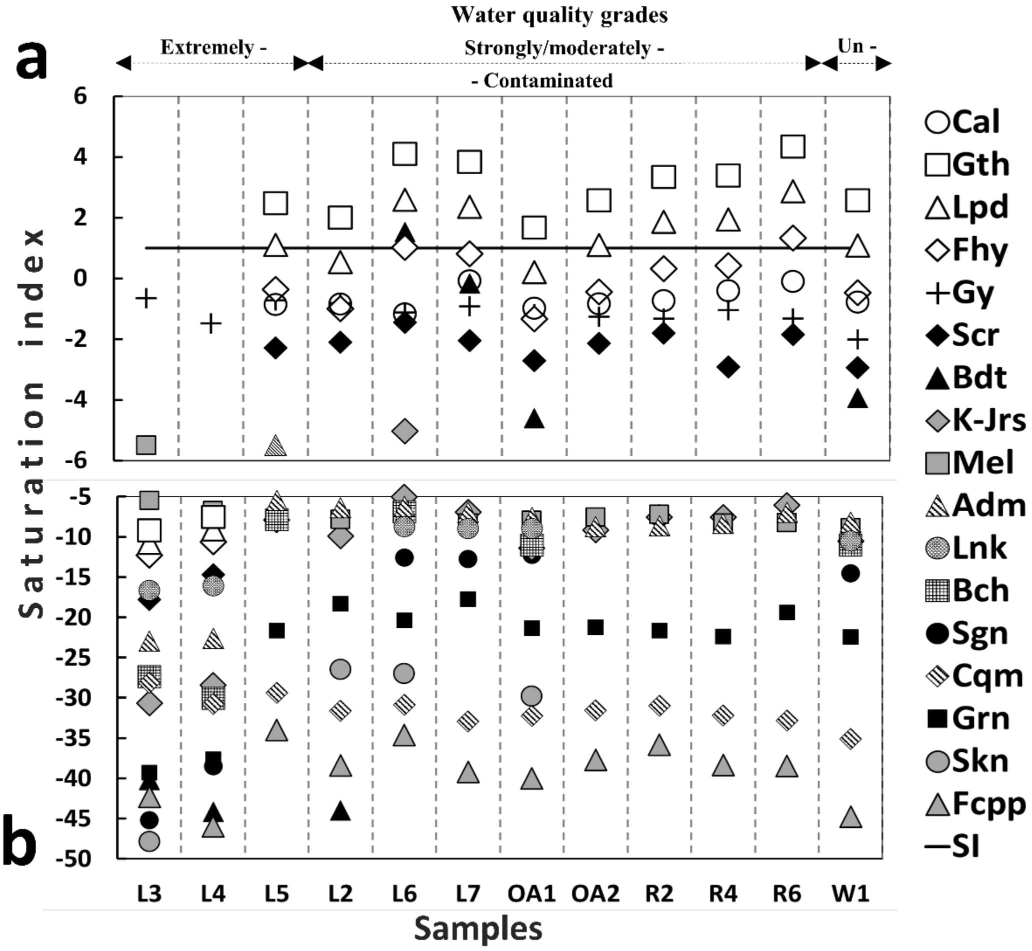

Figure S2. Saturation index estimation of the main mineral phases identified by XRD in samples from AMDW4 when they are in contact with the physicochemical quality of leachate, river, well and spring samples using PHREEQCI (2.15 software).

Table S1. Pearson's correlation coefficient between the physicochemical parameters and ions quantified in the leachates, river, and spring water samples in the study area.

	pH	E_H	DO	EC	Ca	Mg	Na	K	Cl^-	HCO_3^-	CO_3^{2-}	SO_4^{2-}	NO_3^-	Si	As	Al	Cu	Fe	Pb	Sr	Zn	Hg	Tl	Sb	Se
pH	1																								
E_H	−0.32	1																							
DO	0.00	0.11	1																						
EC	−0.90	0.31	−0.17	1																					
Ca	−0.49	0.18	−0.36	0.71	1																				
Mg	0.00	0.05	−0.21	0.26	0.54	1																			
Na	−0.45	0.10	−0.21	0.56	0.66	0.06	1																		
K	−0.14	0.09	−0.25	0.17	0.41	−0.11	0.71	1																	
Cl^-	−0.38	−0.07	−0.02	0.51	0.43	0.11	0.64	0.23	1																
HCO_3^-	0.80	−0.21	−0.08	−0.76	−0.23	0.22	−0.49	−0.14	−0.59	1															
CO_3^{2-}	0.54	−0.27	−0.16	−0.46	0.05	−0.17	0.15	0.44	−0.28	0.45	1														
SO_4^{2-}	−0.45	0.05	−0.25	0.67	0.87	0.39	0.82	0.54	0.56	−0.41	0.17	1													
NO_3^-	−0.62	0.10	−0.06	0.58	0.19	−0.27	0.27	0.02	0.27	−0.63	−0.26	0.16	1												
Si	−0.29	0.54	−0.08	0.30	0.38	0.02	0.36	0.30	−0.24	0.00	0.13	0.28	0.07	1											
As	−0.11	−0.11	−0.02	0.20	0.49	0.26	0.65	0.29	0.45	−0.04	0.21	0.56	-0.16	0.21	1										
Al	-0.82	0.35	-0.10	0.91	0.62	0.20	0.34	-0.06	0.38	-0.66	-0.50	0.50	0.53	0.33	0.08	1									
Cu	−0.81	0.37	−0.15	0.91	0.66	0.21	0.38	0.03	0.38	−0.65	−0.45	0.54	0.54	0.35	0.07	0.99	1								
Fe	−0.85	0.37	−0.19	0.90	0.55	0.05	0.35	−0.04	0.35	−0.74	−0.47	0.46	0.59	0.35	−0.01	0.96	0.95	1							
Pb	−0.34	−0.58	−0.19	0.39	0.31	−0.07	0.30	0.05	0.49	−0.31	−0.14	0.35	0.38	−0.17	0.08	0.40	0.38	0.37	1						
Sr^{2+}	−0.17	−0.17	−0.29	0.29	0.54	0.26	0.72	0.67	0.24	−0.12	0.39	0.73	−0.04	0.25	0.50	−0.03	0.00	−0.01	0.17	1					
Zn	−0.78	0.35	−0.20	0.91	0.68	0.25	0.39	0.02	0.38	−0.61	−0.44	0.55	0.53	0.36	0.10	0.99	0.99	0.94	0.39	0.02	1				
Hg	−0.25	0.47	0.09	0.23	0.08	0.14	0.16	−0.04	0.00	−0.19	−0.29	0.04	0.11	0.29	0.08	0.08	0.07	0.15	−0.43	0.16	0.07	1			
Tl	−0.72	0.23	−0.22	0.84	0.64	0.21	0.28	−0.07	0.32	−0.56	−0.38	0.52	0.54	0.29	−0.01	0.96	0.96	0.92	0.47	−0.04	0.96	−0.02	1		
Sb	−0.38	−0.41	−0.24	0.38	0.18	−0.20	0.42	0.18	0.58	−0.61	−0.10	0.39	0.34	−0.37	0.06	0.25	0.24	0.36	0.68	0.27	0.22	−0.27	0.29	1	
Se	−0.23	−0.24	−0.27	0.30	0.03	0.12	0.02	−0.26	0.41	−0.42	−0.28	0.05	0.57	−0.38	−0.22	0.25	0.24	0.32	0.39	−0.10	0.24	−0.13	0.29	0.53	1

Red and blue numbers have significant positive and negative correlations, respectively ($p < 0.05$).

Table S2. Eigenvectors from principal component analysis for the different types of water samples collected in the study area (with transformed variables to square root values).

	Eigenvectors (Ev_{ij})				
**Variable$^{1/2}$ (var_j)**	**PC1**	**PC2**	**PC3**	**PC4**	**PC5**
pH	−0.278	0.092	0.047	0.182	0.021
E_H	0.081	−0.101	−0.432	−0.179	−0.016
DO	−0.07	−0.132	−0.08	−0.293	−0.266
EC	0.312	−0.021	−0.051	−0.006	−0.062
Ca	0.237	0.237	−0.101	0.252	−0.012
Mg	0.062	0.107	−0.128	0.522	−0.4
Na	0.201	0.333	0.019	−0.226	−0.043
K	0.066	0.365	−0.023	−0.252	0.231
Cl^-	0.187	0.106	0.231	−0.116	−0.37
HCO_3^-	−0.244	0.104	−0.109	0.36	0.092
CO_3^{2-}	−0.124	0.323	0.028	0.013	0.376
SO_4^{2-}	0.228	0.313	0.002	0.074	−0.05
NO_3^-	0.192	−0.163	0.115	−0.23	0.18
Si	0.096	0.11	−0.389	−0.055	0.307
As	0.076	0.332	−0.035	0.01	−0.287
Al	0.295	−0.136	−0.083	0.11	0.05
Cu	0.297	−0.11	−0.092	0.107	0.086
Fe	0.293	−0.148	−0.061	0.012	0.115
Pb	0.156	0.018	0.375	0.132	0.142
Sr	0.088	0.415	0.022	−0.07	−0.037
Zn	0.296	−0.098	−0.091	0.144	0.08
Hg	0.042	−0.016	−0.3	−0.251	−0.353
Tl	0.282	−0.126	−0.035	0.201	0.157
Sb	0.149	0.044	0.421	−0.132	0.012
Se	0.108	−0.161	0.326	0.075	−0.108
Eigenvalue (Eg)	9.773	4.06	3.305	1.866	1.539
Variance (%)	39.1	16.3	13.2	7.5	6.2
Cumulative (%)	39.1	55.4	68.6	76.1	82.2

Table S3. Water quality classification considering the PCA values calculated with physicochemical parameters and chemical compositions of aqueous solution samples.

$Samples_{sk}$	PCA_{sk} value	Water quality grades
**L3**	11.70	Extremely contaminated
**L4**	6.28	
**L5**	5.21	
**OA1**	4.24	Strongly contaminated
**L2**	3.98	
**L7**	3.71	
**R3**	3.49	
**R5**	3.48	
**R4**	3.17	Moderately/strongly contaminated
**L6**	2.96	
**OA2**	2.70	
**R6**	2.63	
**R2**	2.62	
**L1**	2.49	
**R1**	2.28	Slightly/moderately contaminated
**CA**	2.08	
**GA**	1.79	
**W1**	1.61	Uncontaminated
**W2**	1.51	
**EG**	1.40	
**ES**	1.38	

Table S4. Proposed reactions involved in the oxidation of primary sulfide minerals and precipitation or dissolution reactions that produce the SMPs that were identified by XRD in the waste and sediment samples collected from AMDW4 [7,14,52].

Symbol		Chemical reaction of mineral sulfide oxidation in the AMWD weathering		No.	Reference
L	Lautite	$CuAsS$			
		$CuAsS + 5.25O_2 + 1.5H_2O = H_2AsO_4^{-} + 2SO_4^{2-} + Cu^{+} + H^{+}$		1	This study
Ccp	Chalcopyrite	$CuFeS_2$			
		$CuFeS_2 + O_2 + 4H^{+} = Cu^{2+} + Fe^{2+} + 2S + 2H_2O$		2	[7]
		$CuFeS_2 + 4Fe^{3+} = Cu^{2+} + 5Fe^{2+} + 2S$		3	[7]
Cct	Chalcocite	Cu_2S			
		$Cu_2S + 2O_2 = 2Cu^{+} + SO_4^{2-}$		4	This study
Sp	Sphalerite	ZnS			
		$ZnS + 2O_2 = Zn^{2+} + SO_4^{2-}$		5	[14]
Gtn	Gratonite	$PbS_2As_2S_3$			
		$PbS_2As_2S_3 + 13O_2 + 2H_2O = Pb^{2+} + 2H_2AsO_4^{2-} + 5SO_4^{2-}$		6	This study
Py	Pyrite	FeS_2			
		$FeS_2 + 3.5\ O_2 + H_2O = Fe^{2+} + 2SO_4^{2-} + 2H^{+}$		7	[14]
		$FeS_2 + 1.5\ O_2 + H_2O = Fe^{2+} + S + SO_4^{2-} + 2H^{+}$		8	This study
Apy	Arsenopyrite	$AsFeS$			
		$FeAsS + 3.25O_2 + 1.5H_2O = Fe^{2+} + SO_4^{2-} + H_2AsO_4^{-} + H^{+}$		9	[14]
Rlg	Realgar	As_4S_4			
		$As_4S_4 + 13.75\ O_2 + 4.5\ H_2O = H^{+} + 4H_2AsO_4^{2-} + 4SO_4^{2-}$		10	[52]
Orp	Orpiment	As_2S_3			
		$As_2S_3 + 8.75O_2 + 2.5\ H_2O = H^{+} + 2H_2AsO_4^{2-} + 3SO_4^{2-}$		11	[52]
		**Chemical reaction of precipitation (p) and dissolution (d) of secondary mineral phases**			
Gth	Goethite	$FeO(OH)$			
		$Fe^{3+} + 2H_2O = FeO(OH) + 3H^{+}$	p	12	[14]
		$FeO(OH) + 3H^{+} = Fe^{3+} + 2H_2O$	d	13	[7]
He	Hematite	$Fe_{1.8}H_{0.66}O_3$			
		$Fe^{2+} + 0.25O_2 + H^{+} = Fe^{3+} + 0.5H_2O$		14	[7]
		$1.8Fe^{3+} + 3H_2O = Fe_{1.8}H_{0.66}O_3 + 5.34\ H^{+}$	p	15	[14]
		$Fe_{1.8}H_{0.66}O_3 + 5.34\ H^{+} = 1.8Fe^{3+} + 3H_2O$	d	16	This study
Lpd	Lepidocrocite	$Fe^{+3}O(OH)$			
		$Fe^{3+} + 2H_2O = Fe^{+3}O(OH) + 3H^{+}$	p	17	[52]
		$Fe^{+3}O(OH) + 3H^{+} = Fe^{3+} + 2H_2O$	d	18	[52]
Fhy	Ferrihydrite	$Fe(OH)_3$			
		$Fe^{3+} + 3H_2O = Fe(OH)_3 + 3H^{+}$	p	19	This study
		$Fe(OH)_3 + 3H^{+} = Fe^{3+} + 3H_2O$	d	20	[7]
Cal	Calcite	$CaCO_3$			
		$Ca^{2+} + HCO_3^{-} = CaCO_3 + H^{+}$	p	21	This study
		$CaCO_3 + H^{+} = Ca^{2+} + HCO_3^{-}$	d	22	[7]
		$Ca^{2+} + CO_2 + H_2O = CaCO_3 + 2H^{+}$	p	23	This study
		$CaCO_3 + 2H^{+} = Ca^{2+} + CO_2 + H_2O$	d	24	[7]
Gy	Gypsum	$CaSO_4{\cdot}2H_2O$			
		$Ca^{2+} + SO_4^{2-} + 2H_2O = CaSO_4{\cdot}2H_2O$	p	25	This study
		$CaSO_4{\cdot}2H_2O = Ca^{2+} + SO_4^{2-} + 2H_2O$	d	26	[7]
Scr	Scorodite	$FeAsO_4\ (H_2O)_2$			
		$Fe^{3+} + 2H_2AsO_4^{2-} + 2H_2O = FeAsO_4(H_2O)_2 + 4H^{+}$	p	27	This study
		$FeAsO_4(H_2O)_2 + 4H^{+} = Fe^{3+} + 2H_2AsO_4^{2-} + 2H_2O$	d	28	[52]
Bdt	Beudantite	$Pb\ (Fe_{2.5}Al_{0.5})\ (AsO_4)SO_4(OH)_6$			
		$Pb^{2+} + 2.5Fe^{3+} + 0.5Al^{3+} + AsO_4^{3-} + SO_4^{2-} + 6OH^{-} = Pb\ (Fe_{2.5}Al_{0.5})(AsO_4)SO_4\ (OH)_6$	p	29	This study
		$Pb\ (Fe_{2.5}\ Al_{0.5})(AsO_4)SO_4(OH)_6 = Pb^{2+} + 2.5Fe^{3+} + 0.5Al^{3+} + AsO_4^{3-} + SO_4^{2-} + 6OH^{-}$	d	30	[52]
Pb-Jrs	Plumbojarosite	$(Pb_{0.43}K_{0.14})Fe_3(SO_4)_2(OH)_6$			
		$0.43Pb^{2+} + 0.14K^{+} + 3Fe^{3+} + 2SO_4^{2-} + 6H_2O = Pb_{0.43}K_{0.14}Fe_3(SO_4)_2(OH)_6 + 6H^{+}$	p	31	This study
		$Pb_{0.43}K_{0.14}Fe_3(SO_4)_2(OH)_6 + 6H^{+} = 0.43Pb^{2+} + 0.14K^{+} + 3Fe^{3+} + 2SO_4^{2-} + 6H_2O$	d	32	This study
K-Jrs	K-Jarosite	$K_{0.86}(H_3O)_{0.14}Fe_3(SO_4)_2(OH)_6$			

		$3Fe^{3+} + K^{+} + 0.14H_2O + 2SO_4^{2-} + 6H_2O = KFe_3(SO_4)_2(OH)_6 + 6H^{+}$	p	33	[14]
		$KFe_3(SO_4)_2(OH)_6 + 6H^{+} = 3Fe^{3+} + K^{+} + 0.14H_2O + 2SO_4^{2-} + 6H_2O$	d	34	[52]
Mel	Melanterite	$FeSO_4.7H_2O$			
		$Fe^{2+} + SO_4^{2-} + 7H_2O = FeSO_4\ 7H_2O$	p	35	This study
		$FeSO_4\ 7H_2O = Fe^{2+} + SO_4^{2-} + 7H_2O$	d	36	[52]
Adm	Adamite	$Zn_2(AsO_4)(OH)$			
		$2Zn^{2+} + H_2AsO_4^{-} + H_2O = Zn_2(AsO_4)(OH) + 3H^{+}$	p	37	This study
		$Zn_2(AsO_4)(OH) + 3H^{+} = 2Zn^{2+} + H_2AsO_4^{-} + H_2O$	d	38	[52]
Lnk	Lanarkite	$Pb_2(SO_4)O$			
		$2Pb^{2+} + SO_4^{2-} + 0.5O_2 = Pb_2(SO_4)O$	p	39	This study
		$Pb_2(SO_4)O = 2Pb^{2+} + SO_4^{2-} + 0.5O_2$	d	40	[52]
Bch	Brochantite	$Cu_4(SO_4)(OH)_6$			
		$4Cu^{2+} + SO_4^{2-} + 6H_2O = Cu_4(SO_4)(OH)_6 + 6H^{+}$	p	41	This study
		$Cu_4(SO_4)(OH)_6 + 6H^{+} = 4Cu^{2+} + SO_4^{2-} + 6H_2O$	d	42	[52]
Cld	Claudetite	As_2O_3			
		$As_4S_4 + 11O_2 = 2As_2O_3 + 4SO_4^{2-}$	p	43	This study
		$2As_2O_3 + 4SO_4^{2-} = As_4S_4 + 11O_2$	d	44	[52]
Sgn	Segnetite	$PbFe_3(AsO_4)(AsO_3OH)(OH)_6$			
		$Pb^{2+} + 3Fe^{3+} + AsO_4^{3-} + HAsO_4^{2-} + 6H_2O = PbFe_3(AsO_4)(AsO_3OH)(OH)_6 + 6H^{+}$	p	45	This study
		$PbFe_3(AsO_4)(AsO_3OH)(OH)_6 + 6H^{+} = Pb^{2+} + 3Fe^{3+} + AsO_4^{3-} + HAsO_4^{2-} + 6H_2O$	d	46	This study
Cqm	Coquimbite	$Fe_{1.54}Al_{0.46}(SO_4)_3{\cdot}9H_2O$			
		$1.54Fe^{3+} + 0.46Al^{3+} + 3SO_4^{2-} + 9H_2O = Fe_{1.54}Al_{0.46}(SO_4)_3{\cdot}9H_2O$	p	47	This study
		$Fe_{1.54}Al_{0.46}(SO_4)_3{\cdot}9H_2O = 1.54Fe^{3+} + 0.46Al^{3+} + 3SO_4^{2-} + 9H_2O$	d	48	[52]
Grn	Guerinite	$Ca_5(AsO_4)_2(AsO_3(OH))\ 4H_2O$			
		$5Ca^{2+} + 2AsO_4^{3-} + HAsO_4 + 4H_2O = Ca_5(AsO_4)_2(AsO_3(OH))\ 4H_2O$	p	49	This study
		$Ca_5(AsO_4)_2(AsO_3(OH))\ 4H_2O = 5Ca^{2+} + 2AsO_4^{3-} + HAsO_4 + 4H_2O$	d	50	[52]
Skn	Sarkinite	$Mn_2AsO_4(OH)$			
		$2Mn^{2+} + AsO_4^{3-} + H_2O = Mn_2AsO_4(OH) + H^{+}$	p	51	This study
		$Mn_2AsO_4(OH) + H^{+} = 2Mn^{2+} + AsO_4^{3-} + H_2O$	d	52	This study
Fcpp	Ferricopiapite	$Fe_{4.67}(SO_4)_6(OH)_2{\cdot}20H_2O$			
		$4.67Fe^{3+} + 6SO_4^{2-} + 22H_2O = Fe_{4.67}(SO_4)_6(OH)_2{\cdot}20H_2O + 2H^{+}$	p	53	This study
		$Fe_{4.67}(SO_4)_6(OH)_2{\cdot}20H_2O + 2H^{+} = 4.67Fe^{3+} + 6SO_4^{2-} + 22H_2O$	d	54	[52]

Table S5. SEM-EDS analysis of waste and stream sediment samples: AMWD (a, b), XS1 (c, d), XS2 (e), XS3 (f, g) and XS4 (h). SMPs were assigned by mass balance reconstruction based on chemical composition.

	Spot	Element	Wt (%)	At (%)	Mineral phases associated
a	P1	O	55.0	78.3	Fcpp, Bdt, K-Jrs, Als, Lnk
		S	12.8	9.1	
		Fe	13.7	5.6	
		Si	2.5	2.0	
		Al	2.1	1.8	
		Zn	3.5	1.2	
		Pb	7.4	0.8	
		As	2.2	0.7	
		K	0.8	0.5	
	P2	O	48.7	79.6	Lnk, IOH, Fcpp, Bdt, Qz, Adm, Als
		S	8.6	7.0	
		Fe	10.7	5.0	
		Si	4.0	3.7	
		Pb	24.6	3.1	
		As	1.8	0.6	
		Al	0.5	0.5	
		Zn	1.1	0.5	
b	P3	O	27.5	52.6	Scr, Apy
		Fe	24.9	13.6	
		S	11.2	10.6	
		As	24.2	9.9	
		Si	6.6	7.2	
		Al	4.8	5.4	
		K	0.9	0.7	
	P4	O	44.8	67.3	Fcpp, Scr
		S	22.6	16.9	
		Fe	23.6	10.1	
		Al	3.8	3.4	
		As	4	1.3	
		Si	1.2	1.1	
c	P5	O	25.1	51.7	As-bearing IOH, Fcpp, Adm, Scr
		Fe	59.5	35.2	
		As	3.1	1.4	
		Zn	3.6	1.8	
		Si	4.7	5.5	
		S	2.4	2.5	
		Al	1.6	1.9	
	P6	O	31	44.1	Qz
		Si	69	55.9	
d	P7	O	27.2	55.7	Scr, K-Jrs, Adm, Apy
		Fe	30.6	17.9	
		As	25.9	11.3	
		S	7.8	7.9	
		Si	3.7	4.4	
		Zn	3.8	1.9	
		K	1.1	1.0	
e	P8	O	52.1	75.5	Bdt, Fcpp, IOH, Cal, Qz, Als
		Si	10.6	8.7	
		Fe	16.2	6.7	
		Al	3.9	3.3	
		S	4.1	3.0	
		Pb	8.7	1.0	
		As	2.8	0.9	
		Ca	0.8	0.5	
		K	0.7	0.4	
	P9	O	36.5	64.1	IOH
		Fe	52.9	26.6	

		Si	5.3	5.3	Scr
		Al	1.9	1.9	Cal
		Ca	0.9	0.6	Qz
		S	0.7	0.6	Als
		As	1.5	0.6	Fcpp
		K	0.4	0.3	
f	P10	O	33.8	59.4	
		Fe	37.2	18.7	IOH
		Si	8.5	8.5	Scr
		Al	6.1	6.4	Adm
		As	7.7	2.9	Cal
		Ca	3.4	2.4	Qz
		Zn	2.7	1.2	Als
		K	0.8	0.5	
g	P11	O	52.7	77.2	
		Al	2.5	2.2	Fcpp
		Si	4.4	3.7	Bdt
		S	7.3	5.4	IOH
		K	2.0	1.2	Pb-Jrs
		Ca	0.6	0.4	Cal
		Fe	20.2	8.5	Qz
		As	2.1	0.7	Als
		Pb	8.2	0.9	
h	P12	O	46.4	75.2	
		Fe	21.0	9.8	Fcpp
		S	9.0	7.3	Lnk
		Si	2.7	2.5	Bdt
		Pb	16.4	2.1	IOH
		Al	2.0	1.9	Cal
		K	0.7	0.5	Qz
		Ca	0.6	0.4	Als
		As	1.2	0.4	
